# Deciphering the Anticancer Effects of Baicalein in Human Gastric Cancer Cells: Computational Chemistry, Bioinformatics, Network Pharmacology Insights, and in vitro Experimental Validation

**DOI:** 10.5812/ijpr-171172

**Published:** 2026-06-16

**Authors:** Bai Jing, Ren Hui, Li Xiao

**Affiliations:** 1 Medical Record Room, Xingtai People 's Hospital, Xingtai, China; 2 Department of Pharmacy, Xingtai People 's Hospital, Xingtai, China; 3 Internal Medicine of Traditional Chinese Medicine, Xingtai People 's Hospital, Xingtai, China

**Keywords:** Baicalein, Network Pharmacology, Computational Chemistry, Apoptosis, Bioinformatics

## Abstract

**Background:**

Baicalein, a natural flavone, exhibits multifaceted anticancer potential; however, its molecular mechanisms in gastric carcinoma remain unclear.

**Objectives:**

This study aimed to elucidate the molecular mechanisms of baicalein in human gastric cancer (GC) using an integrated approach combining computational chemistry, network pharmacology, bioinformatics, and in vitro assays.

**Methods:**

Network pharmacology and bioinformatics analyses were conducted to identify hub targets and enriched signaling pathways of baicalein in GC. Molecular docking was then performed to evaluate binding affinities for key proteins, including AKT1, STAT3, and mutant TP53. These in silico findings informed subsequent in vitro validation. The primary outcome was cell viability, assessed by the MTT assay; secondary outcomes included apoptosis, assessed by flow cytometry, and changes in protein expression, assessed by Western blotting.

**Results:**

Density functional theory (DFT) analysis indicated favorable electronic properties of baicalein, with a HOMO-LUMO gap of 3.787 eV, and demonstrated similarity to reference inhibitors targeting mutant TP53, AKT, and STAT3. SwissADME indicated drug-likeness, with no rule violations, a bioavailability of 0.55, high gastrointestinal absorption, and blood-brain barrier permeability. Network pharmacology identified 382 targets. Overlap with 1,001 GC genes yielded 181 common targets, forming a significant protein-protein interaction network with 181 nodes and 641 edges and identifying TP53, AKT1, and STAT3 as hubs. The analysis suggested that baicalein may act as a multitarget modulator affecting the PI3K-Akt, MAPK, and apoptosis pathways. In vitro validation supported these predictions. The MTT assay showed selective, dose-dependent cytotoxicity, with IC_50_ values of 40.25 μM in SGC-7901 cells and 77.95 μM in GES-1 cells. Microscopy confirmed apoptotic morphology, flow cytometry showed increased Annexin V-positive cells consistent with intrinsic apoptosis, and Western blotting revealed downregulation of AKT1 and STAT3 with slight upregulation of mutant TP53, supporting their roles in carcinogenesis.

**Conclusions:**

Baicalein may exert selective anticancer effects in GC through multitarget modulation of the PI3K-Akt, MAPK, and apoptosis pathways, particularly via AKT1, STAT3, and mutant TP53. These findings support its potential as a therapeutic candidate, pending further validation.

## 1. Background

Gastric cancer (GC) is a major global health concern with high incidence and mortality, particularly in East Asia (1). It ranks among the top five cancers and is the third leading cause of cancer-related deaths, accounting for approximately 10% of new cases annually (2). Most patients are diagnosed at advanced stages, with a 5-year survival rate of less than 30%, owing to late detection, biological heterogeneity, and rapid chemoresistance (2). Current treatments, including surgery, platinum-based chemotherapy, and targeted agents such as trastuzumab, have limited long-term efficacy because of toxicity and resistance (3), highlighting the need for novel multitarget therapies.

Natural compounds, particularly flavonoids, are promising anticancer candidates because of their efficacy and safety (4). Baicalein (5,6,7-trihydroxyflavone), derived from *Scutellaria baicalensis*, exhibits antioxidant, anti-inflammatory, antiproliferative, proapoptotic, and antimetastatic effects (5, 6). It modulates multiple oncogenic pathways, induces mitochondrial apoptosis through Bax upregulation and Bcl-2 downregulation, regulates the cell cycle, and inhibits epithelial-mesenchymal transition via TGF-β/Smad4 signaling, thereby reducing GC progression (7). However, the comprehensive multitarget mechanism of baicalein in GC remains unclear.

The shift from the "one drug-one target" model to a multitarget paradigm better reflects the complexity of cancer. Network pharmacology integrates systems biology and network analysis to identify drug-target interactions and key hub genes (8), enabling system-level insights into the effects of baicalein in GC. When combined with computational chemistry, molecular docking, and bioinformatics, these approaches provide structural and genomic validation of predicted targets (9, 10). Integrating these methods with in vitro validation offers a robust framework for elucidating the mechanism of baicalein and bridging predictive findings with clinical relevance.

## 2. Objectives

This study was designed using an integrated workflow in which in silico analyses informed subsequent experimental validation. Computational approaches, including DFT, SwissADME, network pharmacology, and molecular docking, were first performed to identify potential targets, pathways, and molecular interactions of baicalein in gastric cancer. These analyses were exploratory and hypothesis-generating. Final targets were selected based on combined criteria of network centrality and pathway relevance, thereby ensuring a focused and biologically meaningful transition from computational prediction to experimental validation.

Based on these predictions, in vitro experiments were conducted to validate the biological effects. The primary outcome was cell viability, assessed by the MTT assay, which served as the principal indicator of anticancer activity. Secondary outcomes included apoptosis induction, assessed by Annexin V/PI staining; morphological assessment; and protein expression analysis, assessed by Western blotting of AKT1, STAT3, and mutant TP53. These secondary outcomes were used to confirm the mechanistic pathways suggested by the in silico findings. This structured approach ensured a logical transition from computational prediction to experimental validation and guided interpretation of the results.

## 3. Methods

### 3.1. Density Functional Theory Analysis of Baicalein and Reference Molecules

All quantum chemical calculations were performed using Gaussian 16 for electronic structure modeling and quantum simulations (11), and GaussView 6 was used to build structures and visualize results. Geometry optimization of baicalein and the reference molecules COTI-2, a mutant TP53 inhibitor; MK-2206, an AKT inhibitor; and napabucasin, a STAT3 inhibitor and GC positive control, was performed using DFT with the B3LYP exchange-correlation functional and the 6 - 311G basis set to obtain thermodynamically stable configurations. The optimized structures were verified as true minima on the potential energy surface by vibrational frequency analysis, confirming the absence of imaginary frequencies. Advanced computational approaches accounting for nonplanar symmetry elements and theoretically derived force constants ensured accurate structural and electronic characterization. Molecular descriptors based on molecular orbital theory were calculated using Koopmans' theorem.

### 3.2. Pharmacokinetic and Toxicity Profiles

The pharmacokinetics and toxicity of baicalein were evaluated to determine drug-likeness and safety, using SwissADME to assess absorption, distribution, metabolism, and excretion (ADME) properties (12). Developed by the Swiss Institute of Bioinformatics, this tool predicts pharmacokinetic parameters and drug-likeness directly from molecular structure. Toxicity endpoints were further predicted using ProTox-3 (13), which applies machine learning and molecular similarity to classify toxicity, estimate acute oral toxicity (LD_50_), and predict organ-specific effects, including mutagenicity, hepatotoxicity, and carcinogenicity, for early safety assessment.

### 3.3. Prediction of Biological Targets for Baicalein

Potential biological targets of baicalein were retrieved from four complementary databases to ensure comprehensive coverage. Targets were first obtained from the Comparative Toxicogenomics Database, which provides experimentally validated chemical-gene-disease associations (14). Additional targets were identified using the Similarity Ensemble Approach, which predicts protein targets through ligand-based similarity scoring across known bioactive compounds (15); only human genes were retained after excluding nonhuman entries. SwissTargetPrediction was also used to rank human targets based on 2-dimensional and 3-dimensional similarity, with a probability cutoff of at least 10% (16). SuperPred further applied molecular fingerprint similarity and machine-learning classifiers, selecting targets with a probability of at least 50% (17). All targets were compiled in Microsoft Excel, duplicates were removed, and the final list was used for overlap analysis with gastric cancer-associated genes.

### 3.4. Gastric Cancer-Associated Targets

Gastric cancer-related targets were obtained from GeneCards, an integrated compendium of functional, expression, and disease-association data, and were filtered using a GIFtS score of at least 60% (18). The filtered GC genes and baicalein targets were analyzed using Jvenn, which generates interactive Venn diagrams and enables the precise identification and export of overlapping genes with statistical annotation (19). The common genes were subsequently used for protein-protein interaction (PPI) network analysis.

### 3.5. Protein-Protein Interaction Network Generation and Visualization

The overlapping genes were submitted to STRING to construct a PPI network integrating experimental, computational, text-mining, and coexpression data, using a confidence score of at least 90%. Unconnected nodes were removed, and the network was exported to Excel. Visualization and topological analysis were performed using Cytoscape version 3.10.3, an open-source platform for the integration, visualization, and analysis of complex biomolecular networks. Hub genes within the PPI network were identified using the cytoHubba plugin, which implements multiple centrality algorithms, including degree, maximum neighborhood component, and maximal clique centrality, to identify topologically critical nodes. To increase specificity and confidence, only the top three ranked hub genes were selected for in vitro validation. This selection was further supported by enrichment analysis, which demonstrated that these hubs, mutant TP53, AKT1, and STAT3, are critically involved in key cancer-related pathways, including PI3K-Akt, MAPK, and apoptosis.

### 3.6. Gene Ontology and Pathway Enrichment Analysis for Baicalein Targets in Gastric Cancer

Functional enrichment of common targets was performed using ShinyGO 0.85 (20), applying a false discovery rate of 0.05 or less to obtain the top 15 terms for each Gene Ontology (GO) category, including biological process, cellular component, and molecular function, and the top 15 Kyoto Encyclopedia of Genes and Genomes (KEGG) pathways, ranked by combined P value and gene count. Results were visualized as publication-grade bar plots using SRPlot, enabling clear ranking by significance and gene count. An integrated mechanistic network was also constructed to depict the multitarget action of baicalein against gastric cancer, linking hub proteins with enriched GO terms and KEGG pathways.

### 3.7. Differential Gene Expression Analysis Using GEPIA2

The top three hub genes were analyzed for differential expression and survival using GEPIA2 (21), which integrates RNA sequencing data from The Cancer Genome Atlas and Genotype-Tissue Expression. Differential gene expression analysis of mutant TP53, AKT1, and STAT3 used an analysis of variance-based method with |log_2_FC| ≥ 1 and q ≤ 0.01, with expression normalized as log_2_(TPM + 1) from matched TCGA normal and GTEx samples in the STAD cohort. Overall survival and disease-free survival were assessed using a median cutoff of 50% for high and low expression groups, a Cox proportional-hazards model, 95% CI, and month-scale plots.

### 3.8. TIMER Analysis

The immunological influence of the three hub genes within the tumor microenvironment was additionally evaluated using the TIMER database (22). This resource quantifies immune-cell infiltration across TCGA cancers through multiple deconvolution algorithms and provides correlation analyses between gene expression and immune subsets. Default settings were retained to assess associations of mutant TP53, AKT1, and STAT3 with B cells, CD4^+^ and CD8^+^ T cells, dendritic cells, macrophages, and neutrophils.

### 3.9. Molecular Docking

Molecular docking was performed to validate interactions between baicalein (PubChem CID 5281605) and three hub proteins. The ligand structure (.pdb) was obtained from PubChem, whereas protein structures were retrieved from the RCSB Protein Data Bank using PDB IDs 4MZI for mutant TP53, 6HHG for AKT1, and 6NUQ for STAT3 (23-25). Protein preparation in Discovery Studio included the removal of water, ligands, and ions; addition of polar hydrogens; CHARMM charge assignment; and energy minimization. Baicalein was similarly minimized and saved in SDF format. Docking was conducted using CB-Dock2 (26), which applies the CurPocket algorithm for cavity detection and AutoDock Vina scoring. The top five cavities per protein were analyzed, and binding poses were visualized using CB-Dock2 and Discovery Studio to generate 2-dimensional interaction profiles with key residues.

### 3.10. Chemicals, Reagents, and Equipment

Baicalein (≥ 98%, Cat. No. 465119) was purchased from Sigma-Aldrich (St Louis, MO, USA). RPMI-1640 (Cat. No. 11875093), fetal bovine serum (FBS; Cat. No. 10082147), penicillin-streptomycin (Cat. No. 15140122), trypsin-EDTA (Cat. No. 25200072), and phosphate-buffered saline (PBS; Cat. No. 10010023) were obtained from Gibco (Waltham, MA, USA). Primary antibodies, including anti-TP53 (9282, 1:1000), anti-AKT1 (2938, 1:1000), anti-STAT3 (12640, 1:1000), and anti-β-actin (4967, 1:5000), and HRP-conjugated secondary antibodies, including anti-rabbit IgG 7074 and anti-mouse IgG 7076, both 1:5000, were obtained from Cell Signaling Technology (Danvers, MA, USA). The Annexin V-FITC/PI kit (Cat. No. 556547) was obtained from BD Biosciences (San Jose, CA, USA). MTT (M2128), crystal violet (C0775), paraformaldehyde (P6148), acridine orange (A6014), and ethidium bromide (E1510) were obtained from Sigma-Aldrich. RIPA buffer (Cat. No. 9806), inhibitor cocktail (Cat. No. 5872), BCA kit (Cat. No. 23225), and ECL substrate (Cat. No. 34580) were obtained from Thermo Fisher Scientific (Hercules, CA, USA). PVDF membranes (0.45 μm, IPVH00010) were obtained from Merck Millipore. Flow cytometry was performed using FACSCalibur (BD Biosciences), imaging was performed using an Olympus IX71 with a DP72 camera (Olympus Corporation), and absorbance was measured using a Model 680 microplate reader (Bio-Rad).

### 3.11. Cell Culture and Conditions

The antiproliferative potential of baicalein was evaluated in GC cells through a comprehensive set of experiments. Gastric cancer SGC-7901 cells and normal gastric epithelial GES-1 cells were obtained from the Cell Bank of the Chinese Academy of Sciences (Shanghai, China). Cell line identity was confirmed based on supplier authentication reports using short tandem repeat profiling, and cells were regularly monitored for morphology and growth characteristics. Both cell lines were maintained in RPMI-1640 medium containing 10% FBS and 1% penicillin-streptomycin at 37 °C in a humidified incubator with 5% CO_2_.

### 3.12. Standardized Baicalein Treatment and Exposure Conditions

Baicalein (> 98% purity) was dissolved in dimethyl sulfoxide (DMSO) to prepare a 100 mM stock solution, which was stored at -20 °C. Working concentrations of 20, 40, 60, and 80 μM were freshly prepared in complete medium, with DMSO maintained at 0.1% (v/v) or less in all groups, including controls, to avoid cytotoxicity. Cells were treated under identical conditions across assays, including 24-hour incubation, defined seeding density, 37 °C, and 5% CO_2_. Controls received an equal volume of DMSO. All experiments used consistent timing and handling to ensure reproducibility.

### 3.13. Evaluation of Cell Viability

The MTT assay was performed on SGC-7901 and GES-1 cells seeded at 5 × 10^3^ cells/well in 96-well plates. After 24-hour treatment with 0 to 80 μM baicalein, 20 μL of MTT (5 mg/mL) was added for 4 hours. Formazan crystals were dissolved in 150 μL of DMSO, and absorbance was measured at 570 nm. Viability was expressed as a percentage of the control.

### 3.14. Colony Formation Assay

SGC-7901 cells were seeded at 500 cells/well in 6-well plates, treated with 0, 20, 60, or 80 μM baicalein, and cultured for 12 days, with the medium refreshed every 3 days. Colonies were fixed with 4% paraformaldehyde, stained with 0.1% crystal violet, and counted. Colonies containing at least 50 cells were included.

### 3.15. Morphological Analysis

After treatment, SGC-7901 cells seeded at 2 × 10^5^ cells/well were washed with PBS and observed using an inverted phase-contrast microscope (Olympus IX71) at 200× magnification. At least 5 random fields per well were imaged.

### 3.16. Apoptosis Analysis

For Annexin V-FITC/PI staining, SGC-7901 cells were trypsinized, washed with PBS, resuspended in 500 μL of binding buffer, stained with 5 μL each of Annexin V-FITC and PI, incubated for 15 minutes in the dark, and analyzed using FACSCalibur. Early apoptotic cells (Annexin V^+^/PI^-^), late apoptotic cells (Annexin V^+^/PI^+^), and necrotic cells were quantified using CellQuest Pro in triplicate.

For acridine orange/ethidium bromide staining, treated cells were stained with 100 μg/mL each of acridine orange and ethidium bromide for 5 minutes in the dark and examined under fluorescence microscopy (Olympus IX71, 400×). Live cells were green, early apoptotic cells were bright green with chromatin condensation, and late apoptotic or necrotic cells were orange/red. At least 5 fields were examined, and the experiment was repeated three times.

### 3.17. Cell Cycle Analysis

Cells were fixed in 70% ethanol at -20 °C overnight, treated with RNase A (100 μg/mL) for 30 minutes at 37 °C, stained with PI (50 μg/mL) for 30 minutes in the dark, and analyzed by FACSCalibur. DNA content was used to determine the G0/G1, S, and G2/M phases using ModFit LT, with triplicate technical replicates in three independent experiments.

### 3.18. Western Blot Analysis

Cells were lysed using RIPA buffer with protease and phosphatase inhibitors. Proteins were quantified using the BCA assay, and 30 μg of protein was separated by 10% SDS-PAGE, transferred to PVDF membranes, and blocked with 5% nonfat milk in TBST. Membranes were incubated with primary antibodies against mutant TP53, AKT1, STAT3, and β-actin overnight at 4 °C, followed by HRP-conjugated secondary antibodies for 1 hour at room temperature. Bands were detected by ECL, quantified using ImageJ, and normalized to β-actin.

### 3.19. Statistical Analysis

Experiments were performed at least three times (n = 3), with triplicate technical replicates. Data are presented as mean ± SD and were analyzed using GraphPad Prism 8.0 with 2-way analysis of variance and Tukey post hoc test. Normality and variance were assessed using the Shapiro-Wilk and Levene tests, respectively. P < 0.05 was considered statistically significant.

## 4. Results

### 4.1. Electronic Structure of Baicalein Using Density Functional Theory

Density functional theory calculations at the B3LYP/6 - 311G level showed that baicalein had a HOMO energy of -5.736 eV, a LUMO energy of -1.949 eV, and an energy gap (ΔE) of 3.787 eV (Figure 1A), indicating a balance between stability and reactivity. The molecule exhibited moderate electrophilicity (ω = 3.898 eV), low hardness (η = 1.894 eV), and high softness (S = 0.528 eV^-1^), supporting favorable biological interactions. A dipole moment of 2.770 D and the molecular electrostatic potential map (Figure 1B) indicated balanced polarity, with electron-rich oxygen regions suitable for hydrogen bonding, whereas the optimized geometry confirmed a near-planar conjugated structure (Figure 1C).

**Figure 1.**
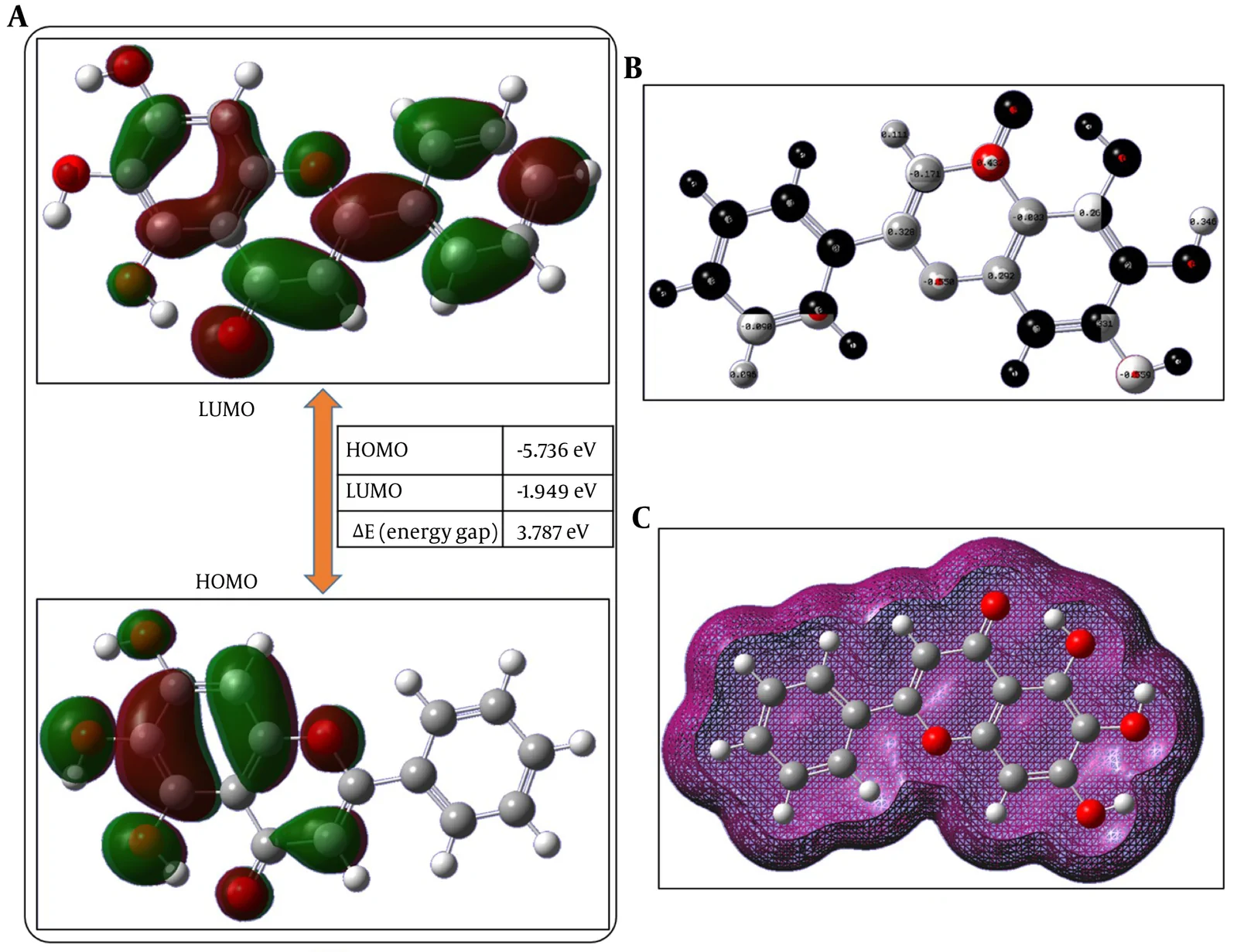
A, Frontier molecular orbital (HOMO–LUMO) distribution of baicalein computed at the B3LYP/6 - 311G level showing HOMO and LUMO energies and energy gap. B, Molecular electrostatic potential (MEP) surface showing electron-rich and electron-deficient regions. C, Optimized molecular geometry illustrating the structural conformation of baicalein.

Comparative DFT analysis demonstrated that baicalein shares electronic characteristics with COTI-2 and MK-2206, particularly similar ΔE values (Figure 2A and B), whereas napabucasin showed a much larger gap of 9.131 eV and lower reactivity (Figure 2C). Baicalein also showed the highest electrophilicity index among all molecules and a dipole moment comparable to that of MK-2206, indicating similar charge distribution and interaction potential (Table 1). In addition, baicalein shares a planar polyaromatic scaffold, a conjugated carbonyl system, and multiple hydrogen-bond donor and acceptor sites with the reference compounds, supporting its ability to interact with AKT1, STAT3, and mutant TP53. The polyphenolic nature of baicalein provides additional antioxidant and metal-chelating properties that are not present in the synthetic references and may contribute to its multitarget profile.

**Table 1.** Electronic Parameters Derived from Density Functional Theory Analysis for Baicalein and Reference Molecules ^a^

Parameter (Symbol)	Baicalein	COTI-2	MK-2206	Napabucasin
**E_HOMO_**	-5.736 eV	-5.39058 eV	-5.58759 eV	-9.54140 eV
**E_LUMO_**	-1.949 eV	-1.35594 eV	-1.66479 eV	-0.41008 eV
**Energy gap (ΔE)**	3.787 eV	4.03463 eV	3.92279 eV	9.13132 eV
**Ionization potential (IE)**	5.736 eV	5.39058 eV	5.58759 eV	9.54140 eV
**Electron affinity (EA)**	1.949 eV	1.35594 eV	1.66479 eV	0.41008 eV
**Absolute electronegativity (χ)**	3.843 eV	3.37326 eV	3.62619 eV	4.97574 eV
**Global hardness (η)**	1.894 eV	2.01732 eV	1.96140 eV	4.56566 eV
**Global softness (S)**	0.528 eV^-1^	0.24785 eV^-1^	0.25492 eV^-1^	0.10951 eV^-1^
**Electrophilicity index (ω)**	3.898 eV	2.82030 eV	3.35190 eV	2.71132 eV
**Dipole moment (μ), Debye**	2.770	4.1830	2.7935	3.9406
**Quadrupole moment (Qxx), Debye·Å**	-83.741	-146.9609	-198.9903	-88.3317
**Quadrupole moment (Qyy), Debye·Å**	-115.864	-151.9740	-168.4742	-118.6259
**Quadrupole moment (Qzz), Debye·Å**	-115.962	-151.8943	-173.2419	-106.7533
**Quadrupole moment (Qxy), Debye·Å**	-1.614	18.7538	9.4641	-6.3744
**Quadrupole moment (Qxz), Debye·Å**	-0.0004	12.9660	4.2915	9.3158
**Quadrupole moment (t/↓), Debye·Å**	-0.0002	-14.2534	2.2165	2.4187

^a^ Abbreviations: HOMO, highest occupied molecular orbital; LUMO, lowest unoccupied molecular orbital.

**Figure 2.**
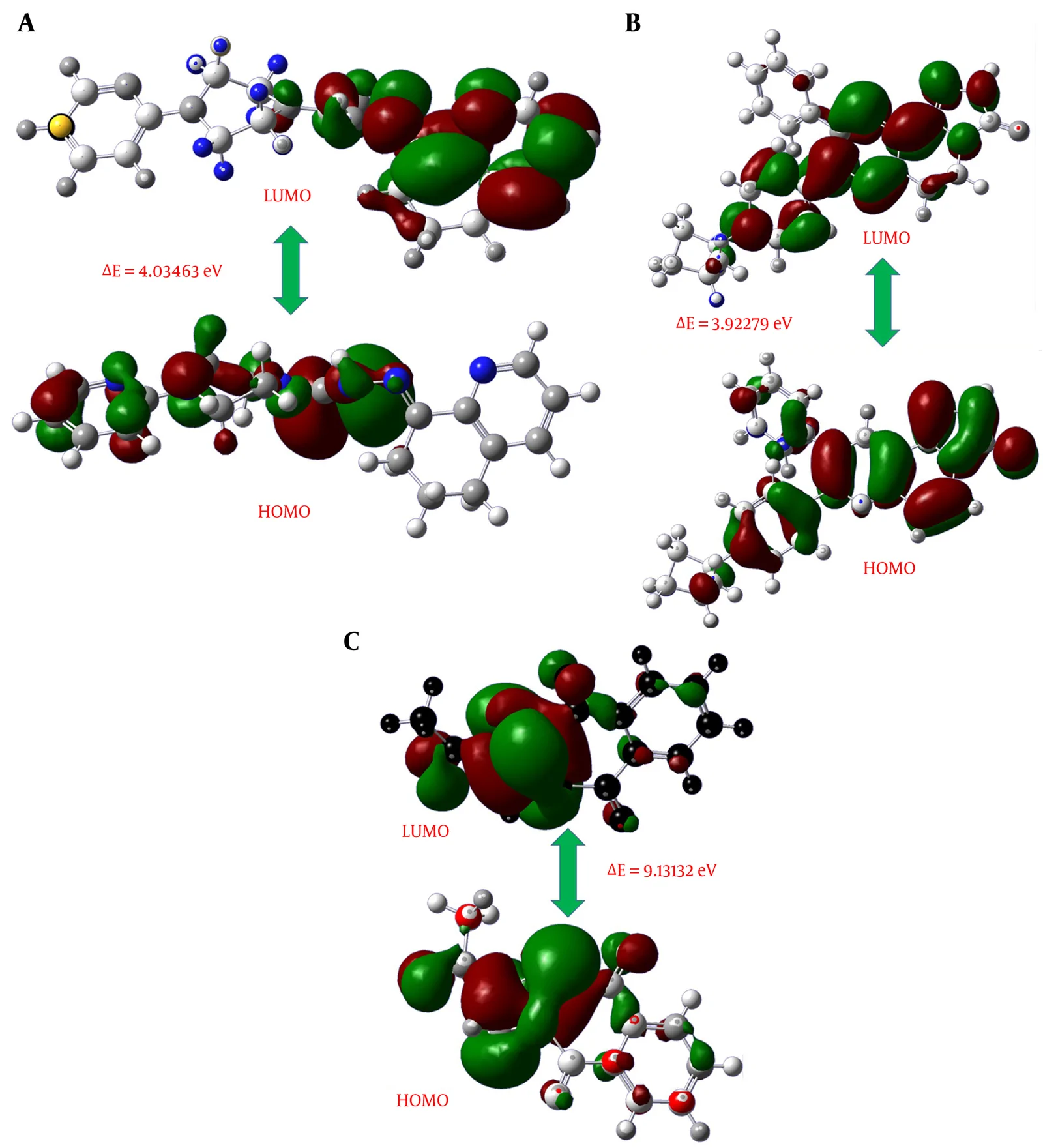
HOMO–LUMO distribution and energy gap analysis of the studied baicalein-based systems. A–C, Frontier molecular orbital maps illustrating the highest occupied molecular orbital (HOMO) and lowest unoccupied molecular orbital (LUMO) distributions for three optimized molecular systems. The corresponding HOMO–LUMO energy gaps were calculated as 4.03463 eV, 3.92279 eV, and 9.3132 eV, respectively. The observed orbital localization and variation in energy gaps provide insight into the electronic stability, charge-transfer behavior, and relative chemical reactivity of the investigated structures.

4.2. Mulliken Charge Distribution, Electron Localization Function, Localized Orbital Locator, and Total Density of States

The Mulliken charge distribution of baicalein showed strong polarization, with oxygen atoms carrying negative charges from -0.40 to -0.60 electrons and several aromatic carbons showing positive charges from +0.20 to +0.40 electrons (Figure 3A). Hydrogen atoms were moderately positive, indicating favorable hydrogen-bonding potential. Similar charge polarization patterns were observed in the reference molecules. In COTI-2, nitrogen atoms showed negative charges from -0.34 to -0.49 electrons, whereas several carbon atoms showed positive charges up to +0.385 electrons, with all hydrogen atoms positively charged (Figure 1A in Supplementary File). MK-2206 displayed a highly negative oxygen atom with a charge of -0.554 electrons and negatively charged nitrogen atoms ranging from -0.41 to -0.59 electrons, along with highly positive carbon centers reaching +0.694 electrons (Figure 1B in Supplementary File). Napabucasin showed the closest pattern to baicalein, with oxygen atoms carrying charges from -0.53 to -0.62 electrons, electron-deficient carbons ranging from +0.49 to +0.57 electrons, and hydrogen atoms from +0.24 to +0.31 electrons (Figure 1C in Supplementary File). Overall, all four molecules exhibited clear charge separation with electron-rich heteroatoms and electron-deficient carbon regions, suggesting similar electrostatic interaction and hydrogen-bonding capabilities with biological targets (Table 2).

**Figure 3.**
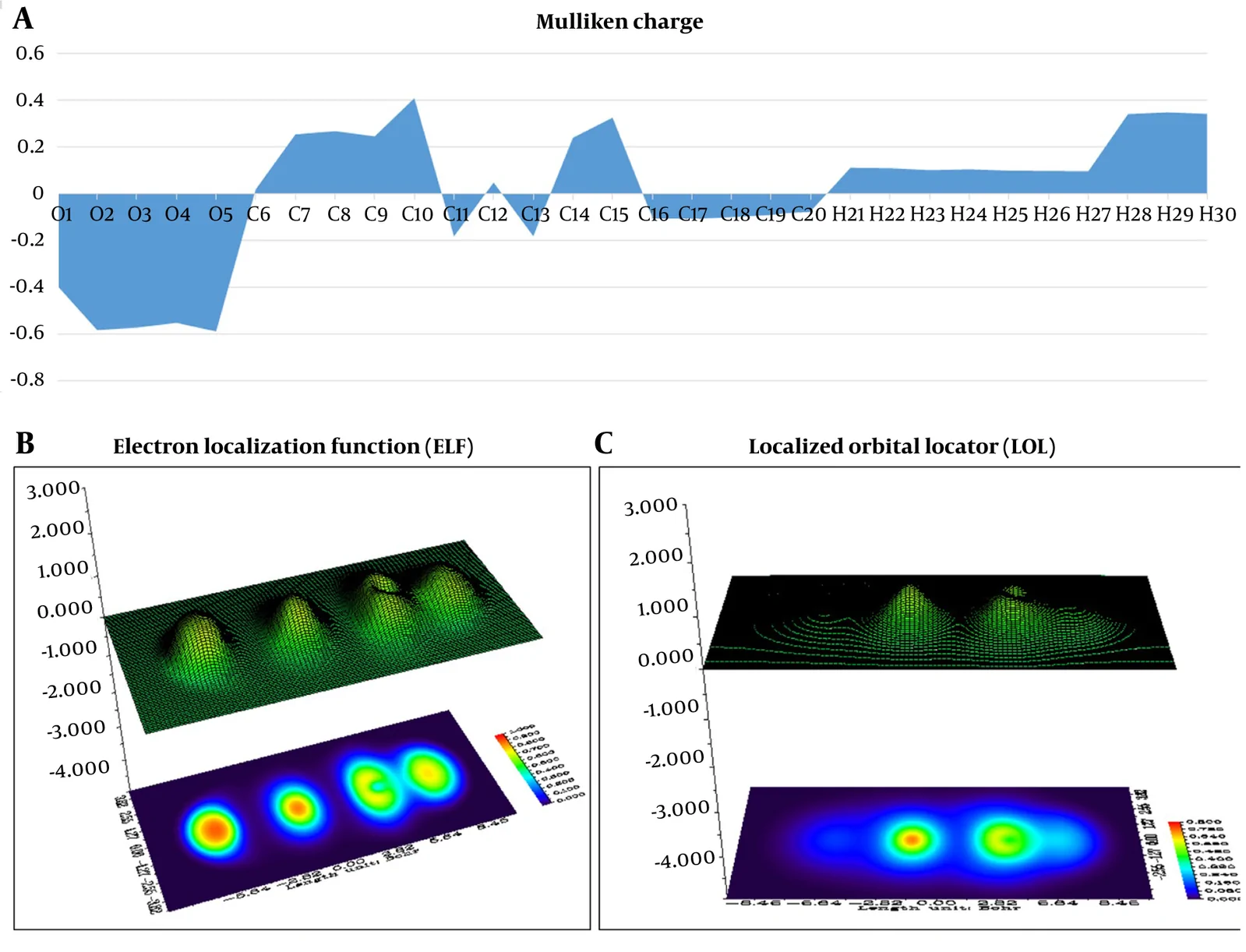
A, Mulliken charge distribution showing atomic charge polarization across the molecule. B, Electron Localization Function (ELF) map indicating regions of localized electron density. C, Localized Orbital Locator (LOL) analysis showing orbital localization in bonding and non-bonding regions.

**Table 2.** ADME and Toxicity Parameters Predicted for Baicalein Using SwissADME and ProTox-III ^a^

Categories and Descriptors	Values
**General properties**	
Molecular formula	C_15_H_10_O_5_
MW	270.24 g/mol
Heavy atoms	20
Aromatic heavy atoms	16
Rotatable bonds	1
**Polarity and surface**	
HBA	5
HBD	3
TPSA	90.9 Å^2^
**Lipophilicity**	
Consensus Log P	2.24
**Solubility**	
ESOL Log S	-4.03
ESOL class	Moderately soluble
**Pharmacokinetics**	
GI absorption	High
BBB permeability	No
P-gp substrate	No
Skin permeability (log Kp)	-5.7 cm/s
**CYP interaction**	
CYP1A2 inhibition	Yes
CYP2D6 inhibition	Yes
CYP3A4 inhibition	Yes
**Drug-likeness**	
Lipinski	0 violations
Veber	0 violations
Ghose	0 violations
Egan	0 violations
Muegge	0 violations
Bioavailability score	0.55
**Medicinal chemistry**	
PAINS alerts	1
Brenk alerts	1
Synthetic accessibility	3.02
**Toxicity predictions**	
Hepatotoxicity	Inactive
Neurotoxicity	Inactive
Cardiotoxicity	Inactive
Immunotoxicity	Inactive
Cytotoxicity	Inactive
Clinical toxicity	Inactive

^a^ Abbreviations: MW, Molecular weight; BBB, blood-brain barrier; CYP, cytochrome P450; ESOL, estimated solubility; GI, gastrointestinal; HBA, hydrogen bond acceptors; HBD, hydrogen bond donors; PAINS, pan-assay interference compounds; P-gp, P-glycoprotein; TPSA, topological polar surface area.

The electron localization function (Figure 3B) showed high localization around oxygen lone pairs, moderate σ-bond localization, and partial π-delocalization across rings. The localized orbital locator (Figure 3C) confirmed localization at bonds and heteroatoms, with low-intensity regions reflecting π-delocalization. These results indicate strong charge polarization, heteroatom-centered localization, and conjugation-driven stability, consistent with molecular electrostatic potential-derived hydrogen-bond acceptor sites and docking interactions with mutant TP53, AKT1, and STAT3. Total density of states analysis (Figure 4A) showed continuous states near the Fermi level dominated by carbon and oxygen p-orbitals, supporting the conjugated system.

**Figure 4.**
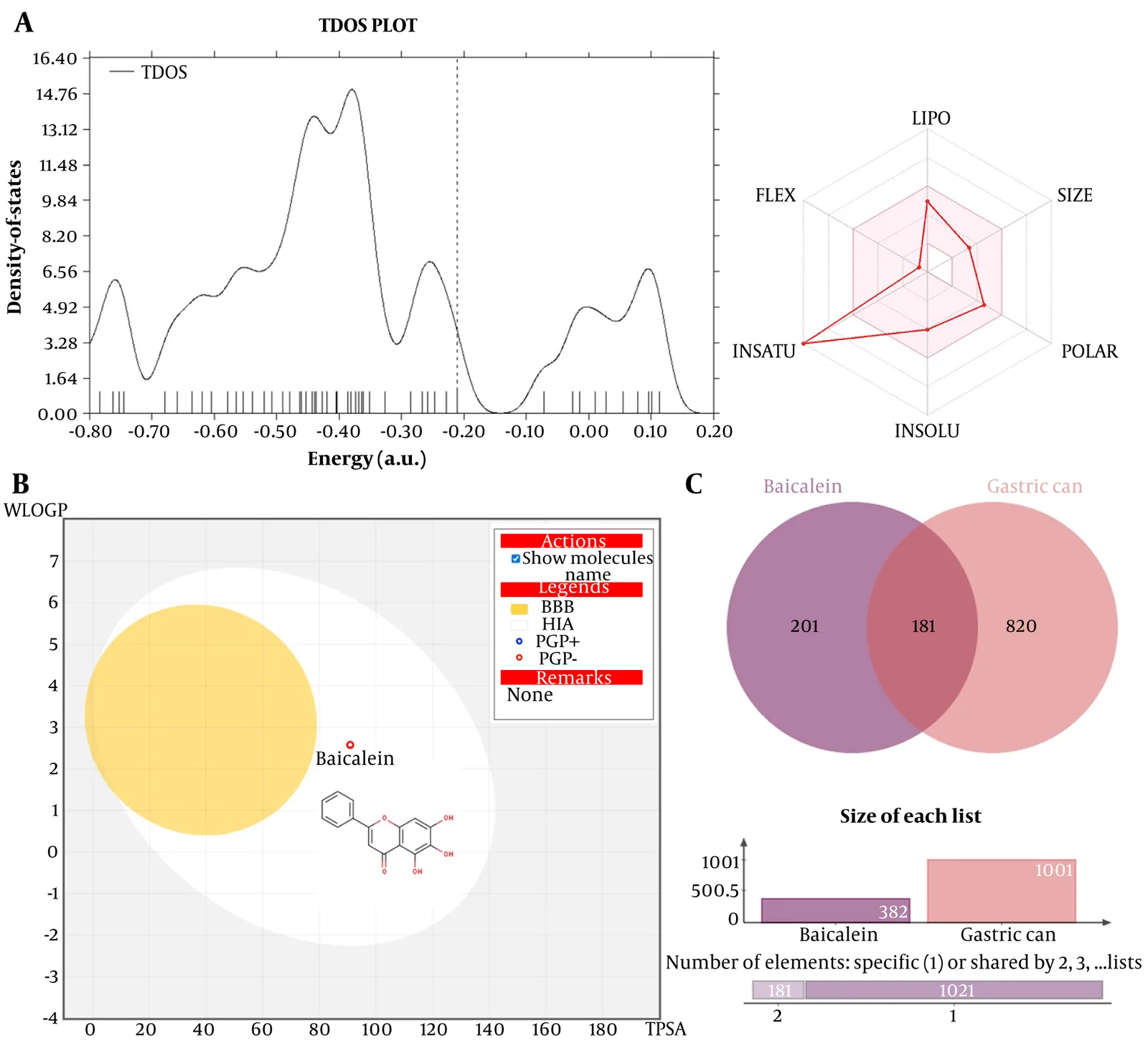
A, Total density of states (TDOS) profile showing electronic energy distribution. B, BOILED-Egg plot predicting gastrointestinal absorption and blood–brain barrier permeability. C, Bioavailability radar illustrating key physicochemical properties. D, Venn diagram showing overlap between baicalein targets and gastric cancer-associated genes.

### 4.3. Predicted Pharmacokinetics and Toxicity of Baicalein

SwissADME showed that baicalein satisfied all five drug-likeness rules, with 0 violations and a bioavailability score of 0.55. Consensus LogP (2.24), TPSA of 90.9 Å^2^, and ESOL of -4.03 indicated balanced lipophilicity and polarity, as well as moderate solubility. High gastrointestinal absorption was predicted, without blood-brain barrier permeation or P-glycoprotein substrate activity, whereas CYP1A2, CYP2D6, and CYP3A4 inhibition suggested interaction potential. The BOILED-Egg plot and bioavailability radar (Figure 4B and C) confirmed favorable pharmacokinetics. ProTox-III predicted no hepatotoxicity, neurotoxicity, cardiotoxicity, immunotoxicity, cytotoxicity, or general clinical toxicity, supporting a favorable safety profile.

The quantum chemical properties of baicalein correlated well with its predicted physicochemical and pharmacokinetic behavior. The moderate ΔE of 3.787 eV and balanced χ and ω indicated suitable chemical stability and reactivity, supporting good drug-likeness and bioavailability. Its moderate dipole moment, high softness, and electron delocalization contributed to balanced polarity, high gastrointestinal absorption, moderate solubility, and stable hydrogen-bonding capacity. The rigid aromatic structure and low rotatable bond count favored pharmacokinetic stability and receptor specificity. These electronic features also aligned with the predicted low-toxicity and non-blood-brain barrier-permeable profile of baicalein.

### 4.4. Target Screening for Baicalein and Gastric Cancer

Target prediction for baicalein across four databases yielded 161 targets from the Comparative Toxicogenomics Database, 75 from Similarity Ensemble Approach after retaining human-only entries, 100 from SwissTargetPrediction at a probability cutoff of at least 10%, and 110 from SuperPred, including 5 confirmed and 105 predicted targets at a probability of at least 50%. After removal of 61 duplicates, 382 unique targets remained. GeneCards yielded 23,769 gastric cancer genes, which were reduced to 1,001 after applying a GIFtS cutoff of at least 60%. Overlap analysis using Jvenn identified 181 common targets (Figure 4D), which were used for subsequent network, enrichment, and validation analyses.

### 4.5. Construction of the Protein-Protein Interaction Network Using STRING and Cytoscape

The 181 common targets were submitted to STRING with a confidence score of at least 90%, generating a highly significant PPI network with 181 nodes, 641 edges, an average node degree of 7.08, an average local clustering coefficient of 0.451, 162 expected edges, and a PPI enrichment P value of less than 1.0 × 10^-16^ (Figure 5A). Topological analysis with cytoHubba using degree, maximal clique centrality, and maximum neighborhood component algorithms (Figure 5B) consistently ranked mutant TP53, AKT1, and STAT3 among the top three hubs across at least two methods (Table 3; Figure 5B and C). These three hub genes were therefore selected for downstream differential expression, survival, immunological, and docking analyses.

**Table 3.** Hub Genes Predicted by the cytoHubba Plugin of Cytoscape Based on Degree, MCC, and MNC Methods ^a^

Degree Rank	Degree Gene	Degree Score	MCC Rank	MCC Gene	MCC Score	MNC Rank	MNC Gene	MNC Score
**1**	TP53	50	1	JUN	10420	1	TP53	48
**2**	AKT1	35	2	TP53	9578	2	AKT1	33
**3**	STAT3	33	3	AKT1	8920	3	STAT3	32
**4**	SRC	31	4	MAPK1	7888	4	SRC	31
**5**	CTNNB1	30	5	STAT3	6715	5	CTNNB1	30
**6**	JUN	27	6	ESR1	5639	6	JUN	27
**7**	ESR1	25	7	SRC	5588	7	MAPK1	25
**7**	MAPK1	25	8	HIF1A	4763	8	ESR1	24
**7**	GRB2	25	9	MAPK3	4240	8	MAPK3	24
**10**	MAPK3	24	10	EGFR	3945	8	GRB2	24

^a^ Abbreviations: MCC, maximal clique centrality; MNC, maximum neighborhood component.

**Figure 5.**
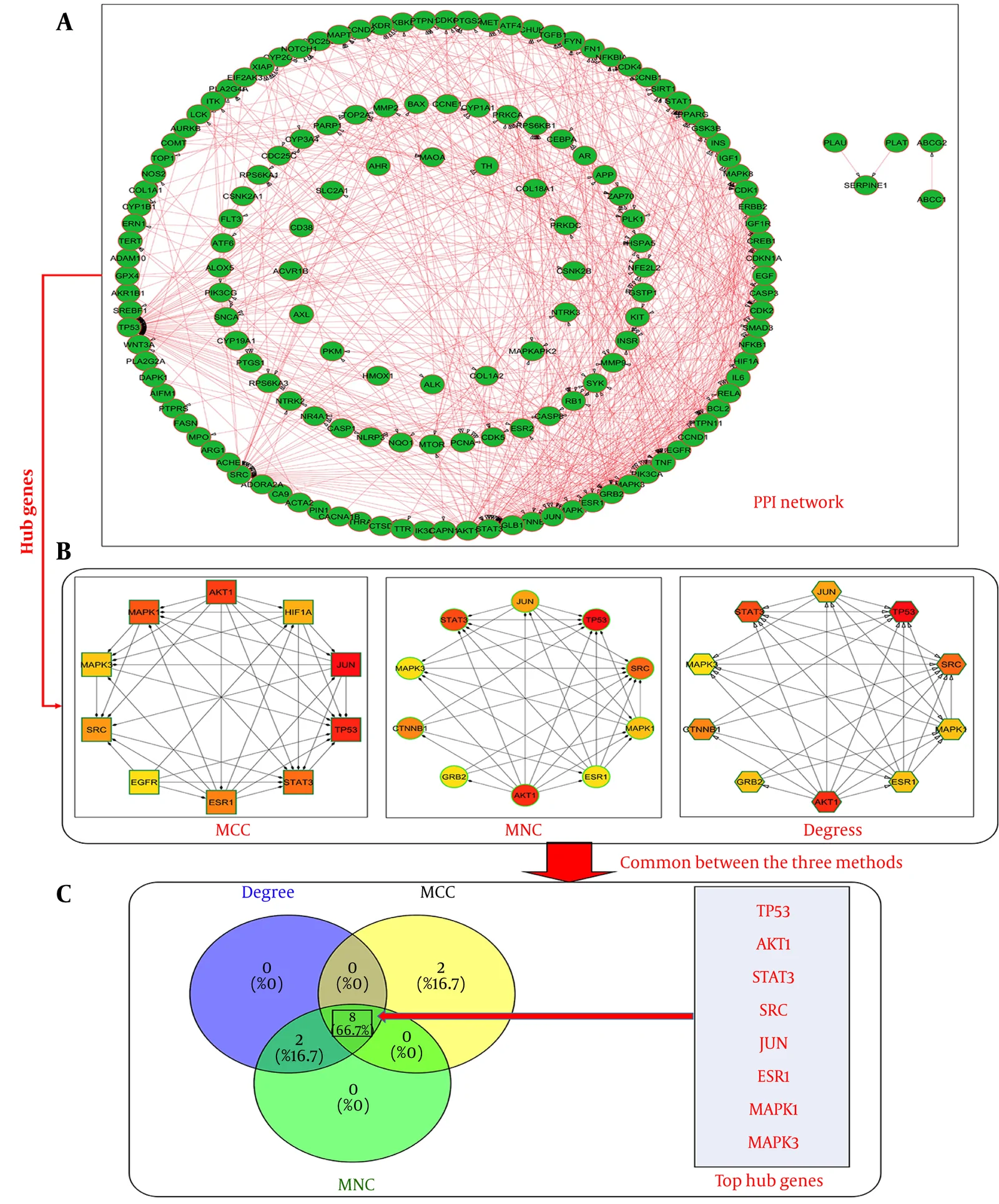
A, Protein–protein interaction (PPI) network of 181 common targets generated using STRING, exhibiting significant connectivity (641 edges; P < 1.0 × 10^-16^) and strong biological interdependence. B, Hub gene identification using cytoHubba algorithms highlighting TP53, AKT1, and STAT3 among the top ranked nodes. C, Subnetwork visualization of key hub genes emphasizing their central regulatory roles in gastric cancerassociated networks.

### 4.6. Gene Ontology and Pathway Enrichment for Baicalein Targets in Gastric Cancer

Functional enrichment of 181 targets using ShinyGO 0.85 with a false discovery rate of less than 0.05 showed strong GO overrepresentation. Biological processes included responses to chemical and oxidative stress, peptidyl-serine phosphorylation, and reactive oxygen species regulation (Figure 6). Cellular components included protein kinase and serine/threonine kinase complexes, membrane rafts, and vesicle lumens. Molecular functions involved serine/threonine kinase activity, protein tyrosine kinase activity, and transcription factor binding. KEGG pathway analysis identified 15 enriched pathways (Figure 7A), with the PI3K-Akt pathway ranked highest, with a gene ratio of 0.2458 and P = 6.81 × 10^-24^, followed by the MAPK pathway, with a gene ratio of 0.2067 and P = 2.73 × 10^-20^, and human papillomavirus infection, with a gene ratio of 0.2179 and P = 1.46 × 10^-20^. Mapping highlighted multiple baicalein targets in the PI3K-Akt axis (Figure 7B), supporting a multitarget mechanism.

**Figure 6.**
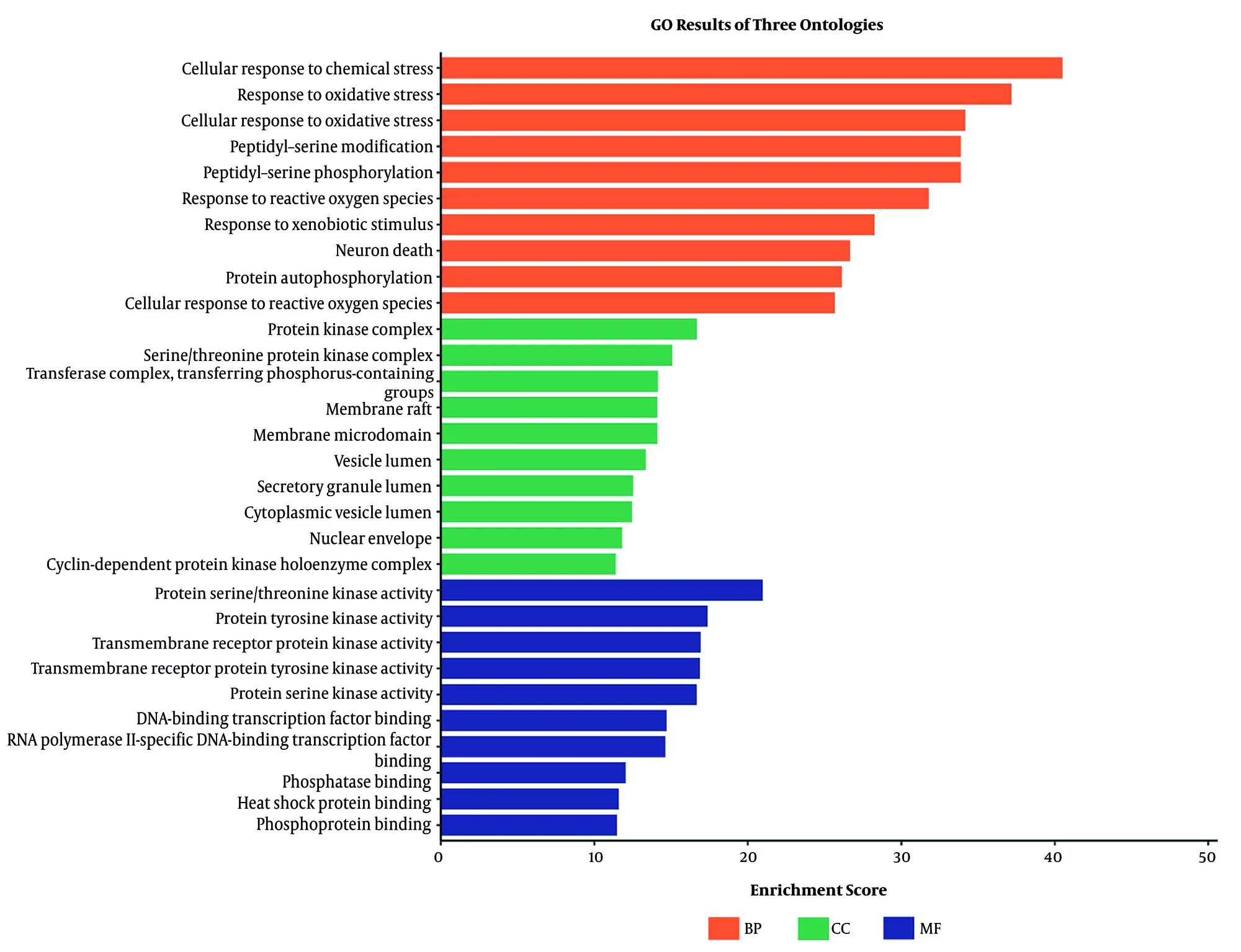
A, Gene Ontology enrichment analysis showing major biological process categories. B, cytoHubba-based hub gene ranking using degree, MCC, and MNC methods. C, Visualization of enriched functional categories and molecular functions.

**Figure 7.**
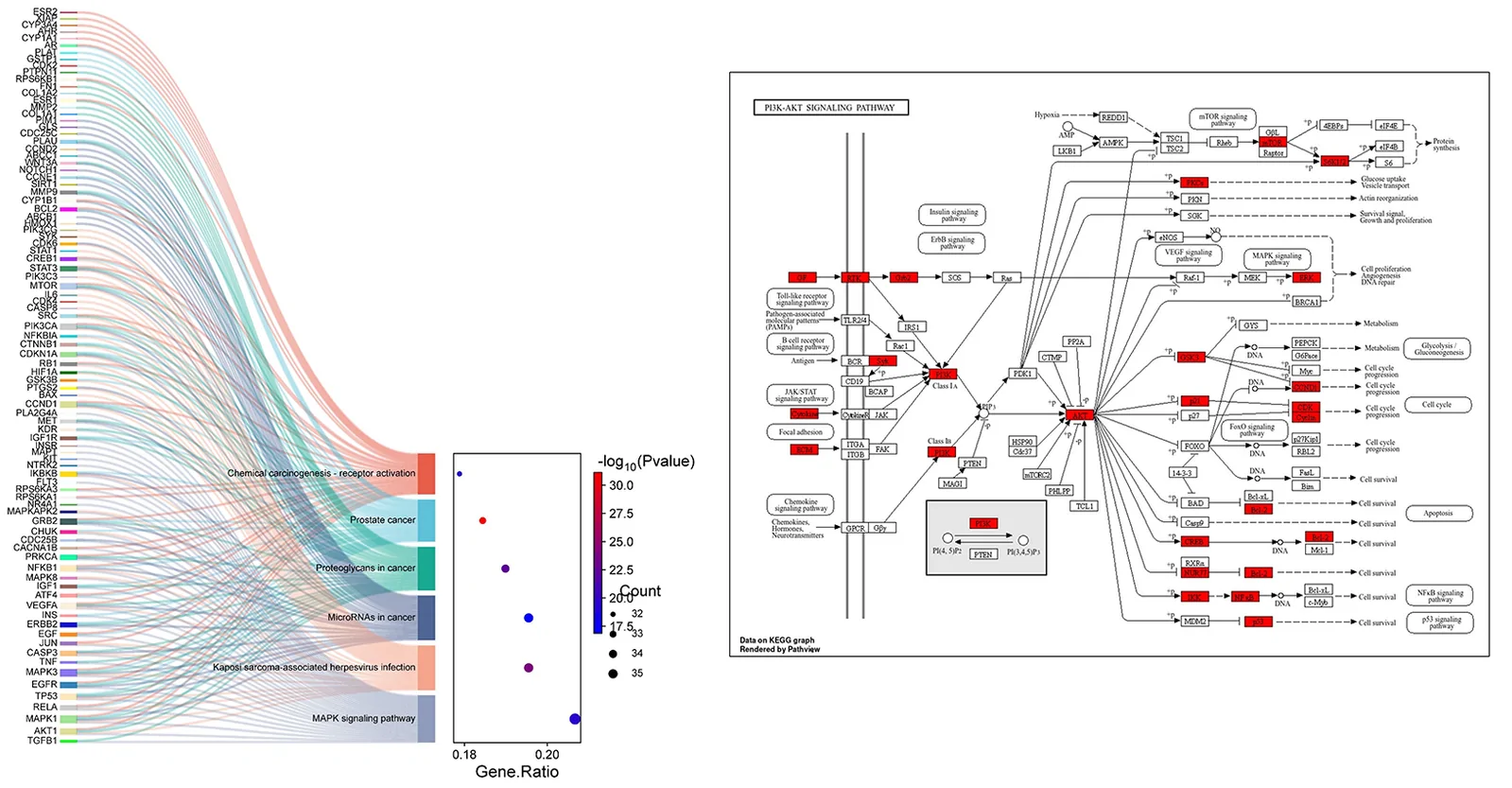
A, KEGG pathway enrichment (Sankey plot) showing association with major signalling pathways. B, Mapping of common targets within the PI3K-Akt signalling pathway

### 4.7. Baicalein-Target Protein-Signaling Pathway Network Construction

The integrative network pharmacology analysis suggested that baicalein may act as a multitarget modulator connected to multiple proteins and pathways (Figure 8). Associations with apoptosis, oxidative stress, and inflammation imply potential polypharmacological effects. Predicted links to the PI3K-Akt, MAPK, and viral infection pathways, including human papillomavirus and cytomegalovirus pathways, indicate possible roles in tumor progression and immune modulation. Dense interactions suggest pathway crosstalk and highlight hub proteins as potentially important regulators, whereas connections to microRNAs, proteoglycans, and lipid metabolism indicate broader biological involvement.

**Figure 8.**
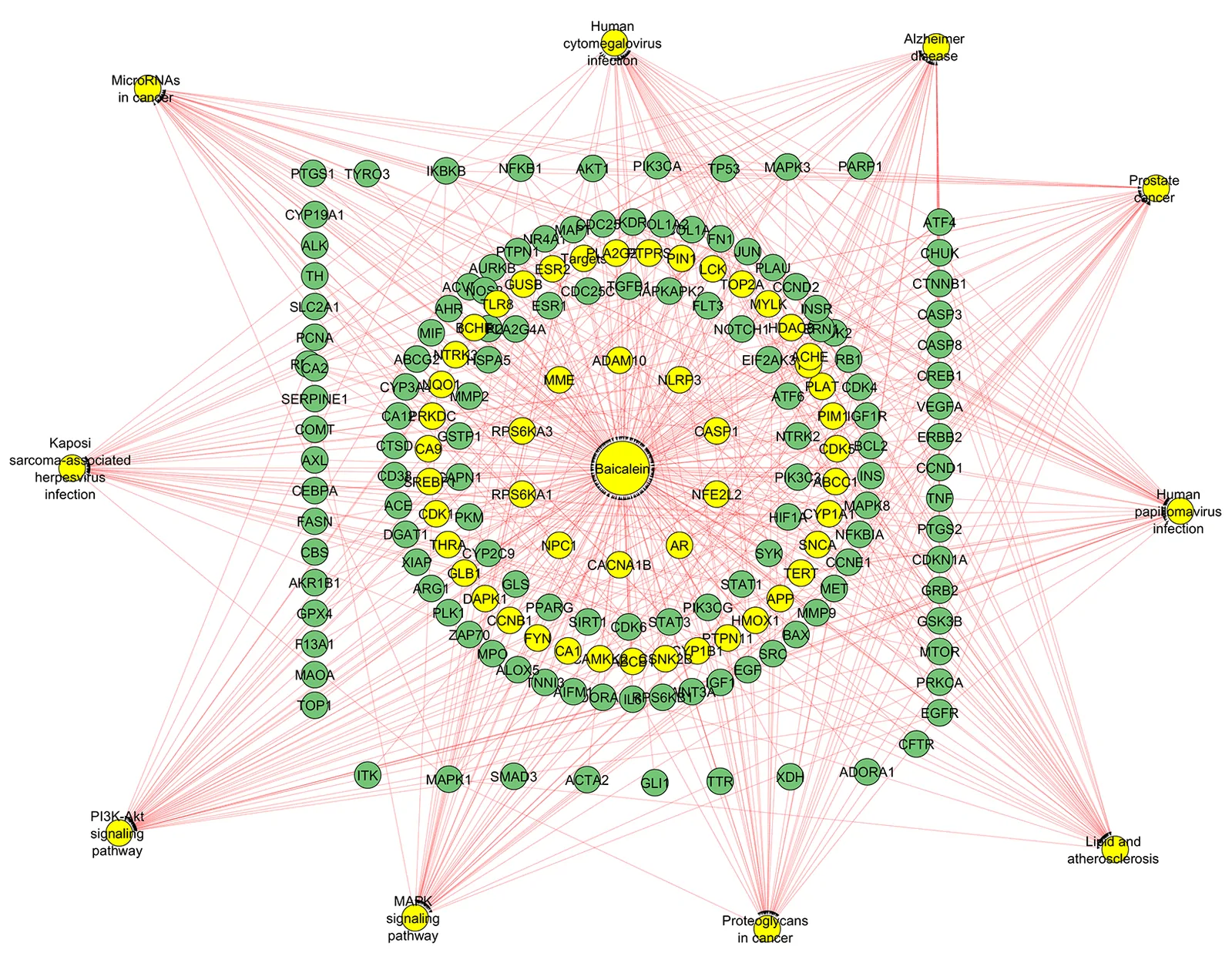
Integrated network pharmacology diagram depicting Baicalein as a central multitarget regulator interacting with key proteins and multiple interconnected signalling pathways, reflecting its polypharmacological mechanism.

### 4.8. Differential Gene Expression of Mutant TP53, AKT1, and STAT3 in Gastric Cancer

Expression profiling of mutant TP53, AKT1, and STAT3 in the STAD dataset using GEPIA2 showed significant upregulation in gastric cancer tissues compared with normal tissues, based on analysis of variance, |log_2_FC| > 1, and q < 0.01 (Figure 9A-C). Kaplan-Meier analysis suggested poorer outcomes with higher expression. Mutant TP53 disease-free survival approached significance (P = 0.054, HR = 0.69), whereas AKT1 and STAT3 overall survival and disease-free survival showed nonsignificant but consistent HR values greater than 1 (Figure 9A-C). These findings suggest potential prognostic relevance of the identified PPI hubs in GC.

**Figure 9.**
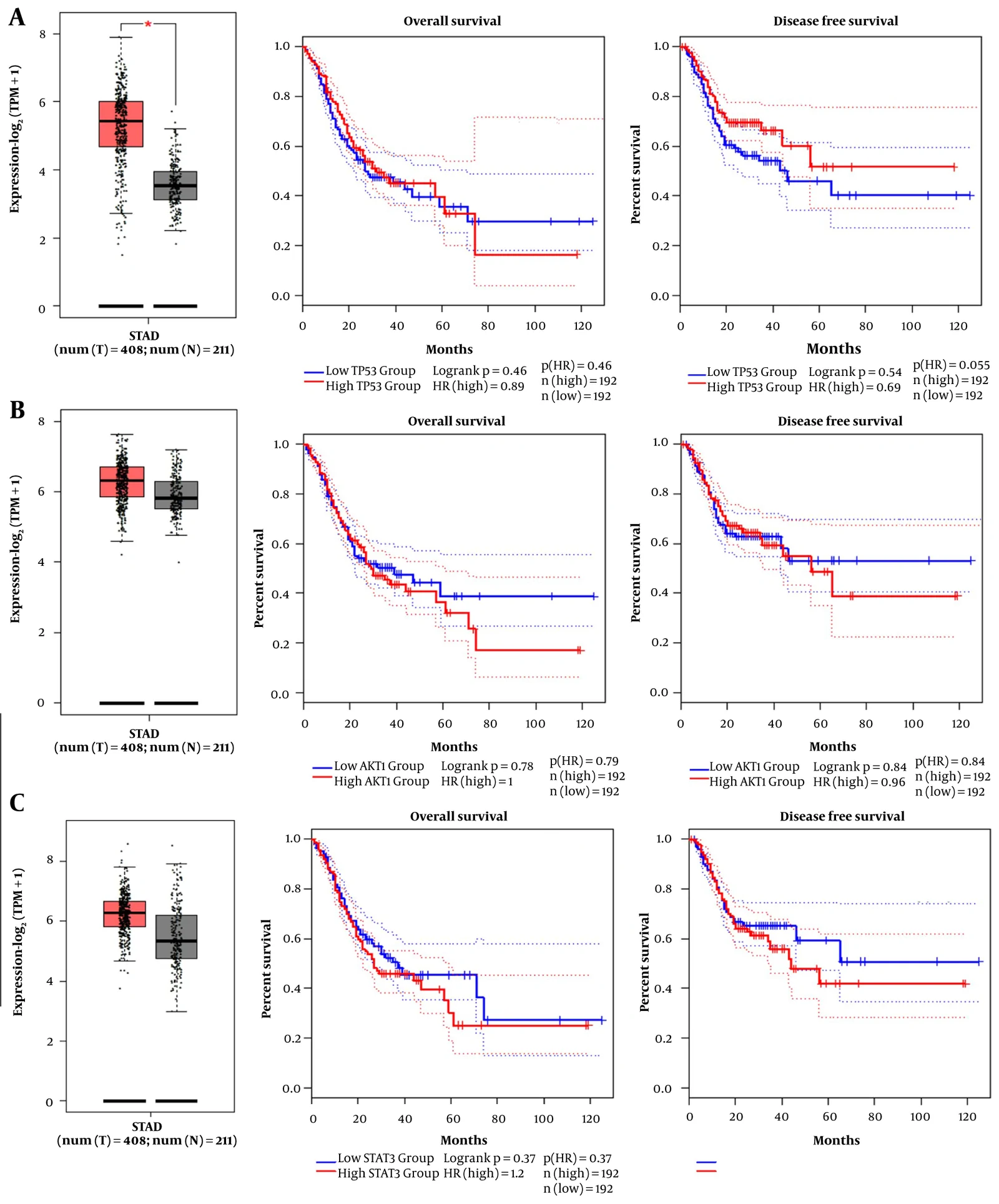
A–C, Differential gene expression analysis (left panels) showing significant upregulation of TP53, AKT1, and STAT3 in gastric cancer tissues compared to normal controls, and corresponding Kaplan–Meier survival curves (right panels) indicating trends toward poorer prognosis with elevated expression, supporting their clinical relevance.

### 4.9. Influence of Mutant TP53, AKT1, and STAT3 on the Gastric Cancer Tumor Microenvironment

The TIMER analysis linked the hub genes to tumor microenvironment modulation (Figure 10). Mutant TP53 exhibited a significant negative partial correlation with B-cell infiltration (partial correlation = -0.115, P = 0.026) and positive associations with CD8^+^ T cells and neutrophils. AKT1 displayed modest negative correlations with CD8^+^ T cells and neutrophils. In contrast, STAT3 showed robust positive correlations with CD4^+^ T cells (partial correlation = 0.297, P < 1 × 10^-8^), macrophages (partial correlation = 0.338, P < 1 × 10^-10^), neutrophils (partial correlation = 0.242, P < 1 × 10^-5^), and dendritic cells (partial correlation = 0.317, P < 1 × 10^-9^), indicating a pronounced influence on immunosuppressive and protumorigenic immune infiltration in gastric cancer.

**Figure 10.**
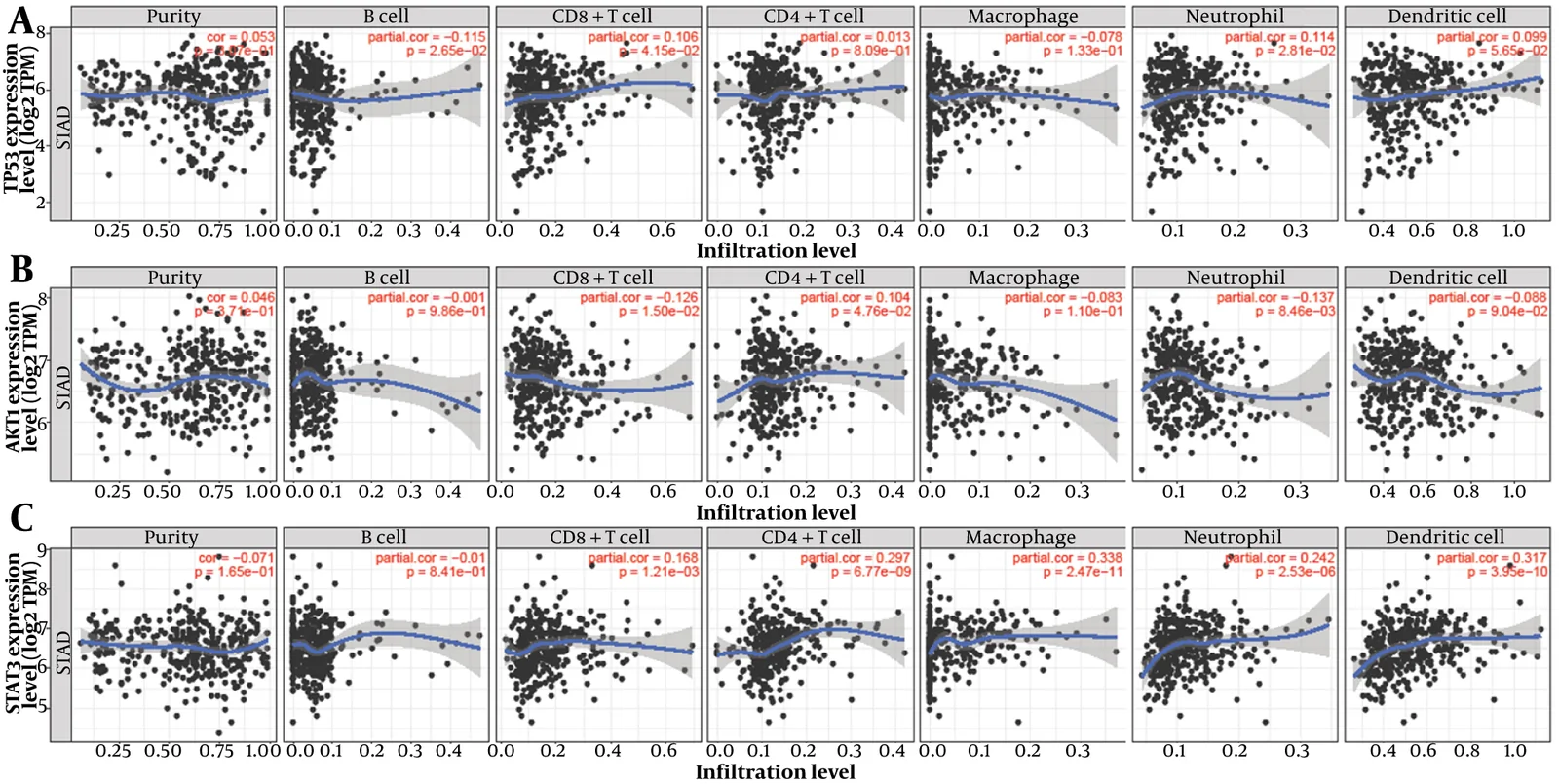
Correlation analysis between hub gene expression and immune cell infiltration demonstrating distinct immunomodulatory roles, with mutant-TP53 showing mixed associations, AKT1 exhibiting modest negative correlations, and STAT3 displaying strong positive correlations with multiple immune cell populations, indicating its prominent involvement in tumor microenvironment regulation.

### 4.10. Molecular Docking-Based Prediction and Relationship with Quantum Chemical Parameters

Blind docking evaluated baicalein binding with mutant TP53 (4MZI), AKT1 (6HHG), and STAT3 (6NUQ) (Tables 4 and 5). For mutant TP53, the best pose, C4, scored -6.6 kcal/mol, with a volume of 116 Å^3^ and center coordinates of -28, -4, and -1. It showed a hydrogen bond/Pi-donor interaction with TYR229, a Pi-anion interaction with GLU224, van der Waals contacts with ASN200, GLU221, GLY199, THR230, THR231, and TYR233, and one unfavorable clash with GLU198 (Figure 11A). Other cavities ranged from -6.3 to -5.2 kcal/mol. AKT1 showed the strongest affinity, with C1 scoring -9.7 kcal/mol, a volume of 6836 Å^3^, and center coordinates of 9, -13, and -12. It showed hydrogen bonds with SER205, THR211, and THR291; a Pi-cation interaction with TRP80; a Pi-anion interaction with ASP292; a Pi-alkyl interaction with LEU210; Pi-sigma interactions with LEU264, LYS268, and VAL270; and van der Waals contacts with ALA212, ASN53, ILE290, LEU213, and TYR272 (Figure 11B). STAT3, with C3 scoring -7.3 kcal/mol, a volume of 479 Å^3^, and center coordinates of 16, 11, and 18, showed hydrogen bonds with ASP369, GLU455, and THR440; Pi-sigma interactions with ASP371, LEU438, and VAL490; and van der Waals contacts with HIS437, HIS457, LEU436, LYS370, and LYS488, indicating SH2-domain engagement (Figure 11C). Overall, baicalein was predicted to bind all three proteins with moderate to high affinity, in the order AKT1 > STAT3 > mutant TP53, through van der Waals contacts, Pi interactions, and hydrogen bonds. These findings support a potential multitarget mechanism consistent with the network analyses, pending experimental validation.

**Table 4.** CB-Dock2-Predicted Docking Scores for Baicalein with Mutant TP53, AKT1, and STAT3

CurPocket	Docking score	Volume (Å^3^)	Center (x, y, z)	Docking size (x, y, z)
**Baicalein-mutantTP53**				
C4	-6.6	116	-28, -4, -1	20, 20, 20
C2	-6.3	151	-20, 9, 7	20, 20, 20
C5	-6.2	104	-17, -19, 8	20, 20, 20
C3	-5.4	121	-15, 1, 21	20, 20, 20
C1	-5.2	170	-33, -8, 9	20, 20, 20
**Baicalein-AKT1**				
C1	-9.7	6836	9, -13, -12	30, 35, 35
C2	-7.6	1777	25, -22, -24	20, 30, 28
C4	-6.6	297	12, 4, -31	20, 20, 20
C5	-6.5	191	2, 2, -4	20, 20, 20
C3	-5.9	501	5, -12, -33	20, 20, 20
**Baicalein-STAT3**				
C3	-7.3	479	16, 11, 18	20, 20, 26
C1	-7.1	830	0, 25, 31	20, 20, 20
C2	-7.0	730	5, 32, 20	20, 20, 20
C4	-6.8	451	-1, 9, 26	20, 20, 20
C5	-5.6	403	-29, -28, 59	20, 20, 20

**Table 5.** CB-Dock2-Predicted Docking Interactions for Baicalein with Mutant TP53, AKT1, and STAT3

Protein	Interaction Type	Residues
**mutant TP53**	Conventional hydrogen bond/Pi-donor	TYR A:229
**mutant TP53**	Pi-anion/attractive charge	GLU A:224
**mutant TP53**	Unfavorable acceptor-acceptor	GLU A:198
**mutant TP53**	van der Waals	ASN A:200, GLU A:221, GLY A:199, THR A:230, THR A:231, TYR A:233
**AKT1**	Attractive charge/Pi-cation	TRP A:80
**AKT1**	Conventional hydrogen bond	SER A:205, THR A:211, THR A:291
**AKT1**	Pi-alkyl/Pi-stacked	LEU A:210
**AKT1**	Pi-anion	ASP A:292
**AKT1**	Pi-sigma	LEU A:264, LYS A:268, VAL A:270
**AKT1**	van der Waals	ALA A:212, ASN A:53, ILE A:290, LEU A:213, TYR A:272
**STAT3**	Conventional hydrogen bond	ASP A:369, GLU A:455, THR A:440
**STAT3**	Pi-sigma	ASP A:371, LEU A:438, VAL A:490
**STAT3**	van der Waals	HIS A:437, HIS A:457, LEU A:436, LYS A:370, LYS A:488

**Figure 11.**
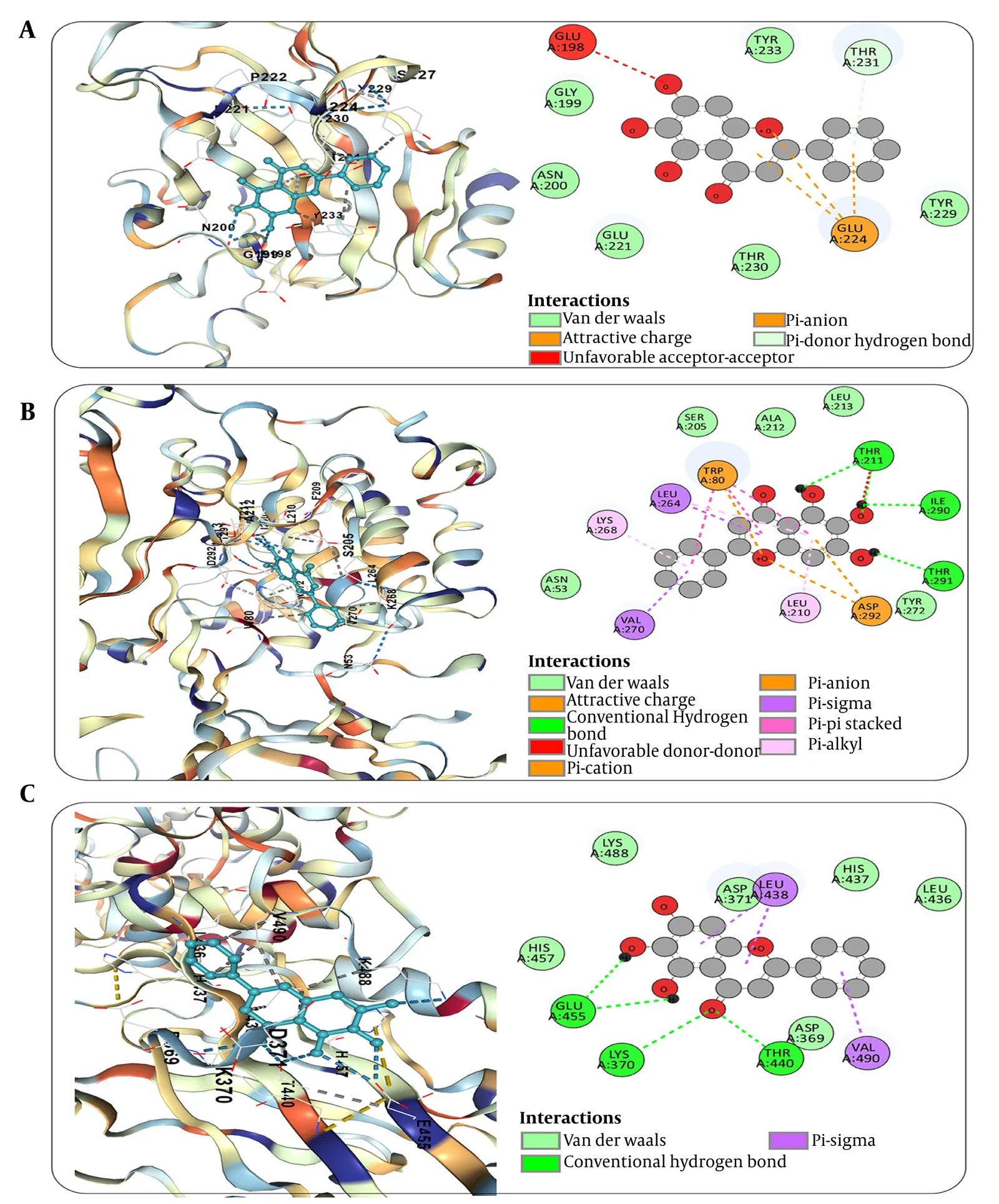
A, Molecular docking of baicalein with mutant-TP53 showing stable binding (−6.6 kcal/mol) via hydrogen bonding, π-interactions, and van der Waals contacts within functional pockets. B, Docking with AKT1 demonstrating the strongest binding affinity (−9.7 kcal/mol), supported by multiple hydrogen bonds and π-based interactions within the kinase domain. C, STAT3 interaction profile (−7.3 kcal/mol) indicating stable binding within the SH2 domain through hydrogen bonding and hydrophobic interactions, collectively supporting a multitarget inhibitory mechanism.

### 4.11. Correlation Between Quantum Chemical Parameters and Baicalein Docking Affinity Against Critical Hub Genes

The DFT-derived quantum descriptors of baicalein closely correlated with its docking behavior against TP53, AKT1, and STAT3. E_HOMO_ = -5.736 eV indicated strong electron-donating ability, supporting charge-transfer interactions with residues including TYR229 and GLU224 in mutant TP53, TRP80 and ASP292 in AKT1, and ASP369 and GLU455 in STAT3 through hydrogen bonding and π-interactions. E_LUMO_ = -1.949 eV and moderate ΔE = 3.787 eV suggested good reactivity and structural adaptability, enabling stable binding within protein pockets, particularly with AKT1, which showed the strongest affinity of -9.7 kcal/mol. IE = 5.736 eV, EA = 1.949 eV, χ = 3.843 eV, and ω = 3.898 eV supported balanced electron donation and acceptance and interactions with both electron-rich and electron-deficient residues. A moderate η of 1.894 eV and high S of 0.528 eV^-1^ indicated sufficient flexibility for efficient electron redistribution during ligand-protein binding, consistent with extensive van der Waals interactions. The dipole moment of 2.770 D favored stable orientation in polar binding regions and strengthened hydrogen bonding with SER205, THR211, THR291, ASP369, GLU455, and THR440, whereas the aromatic flavone core and negative quadrupole moments enhanced π-anion, π-cation, and π-sigma interactions. Collectively, these findings explain the stable binding of baicalein with mutant TP53, AKT1, and STAT3.

### 4.12. Cytotoxic Effects of Baicalein in SGC-7901 Cells

Baicalein exerted potent, dose-dependent antiproliferative effects on SGC-7901 GC cells while sparing normal gastric epithelial GES-1 cells. Treatment with baicalein for 24 hours reduced the viability of SGC-7901 cells, with an IC_50_ of 40.25 ± 3.40 μM (R^2^ = 0.98), whereas GES-1 cells showed markedly higher resistance, with an IC_50_ of 77.95 ± 9.5 μM (R^2^ = 0.97; Figure 12A). This selectivity was further evident in the colony formation assay, in which baicalein progressively suppressed the number and size of colonies formed by SGC-7901 cells (Figure 12B). Quantitative analysis confirmed a clear dose-dependent decline, yielding an IC_50_ of 95.94 ± 20 μM for colony inhibition (R^2^ = 0.91; Figure 12C).

**Figure 12.**
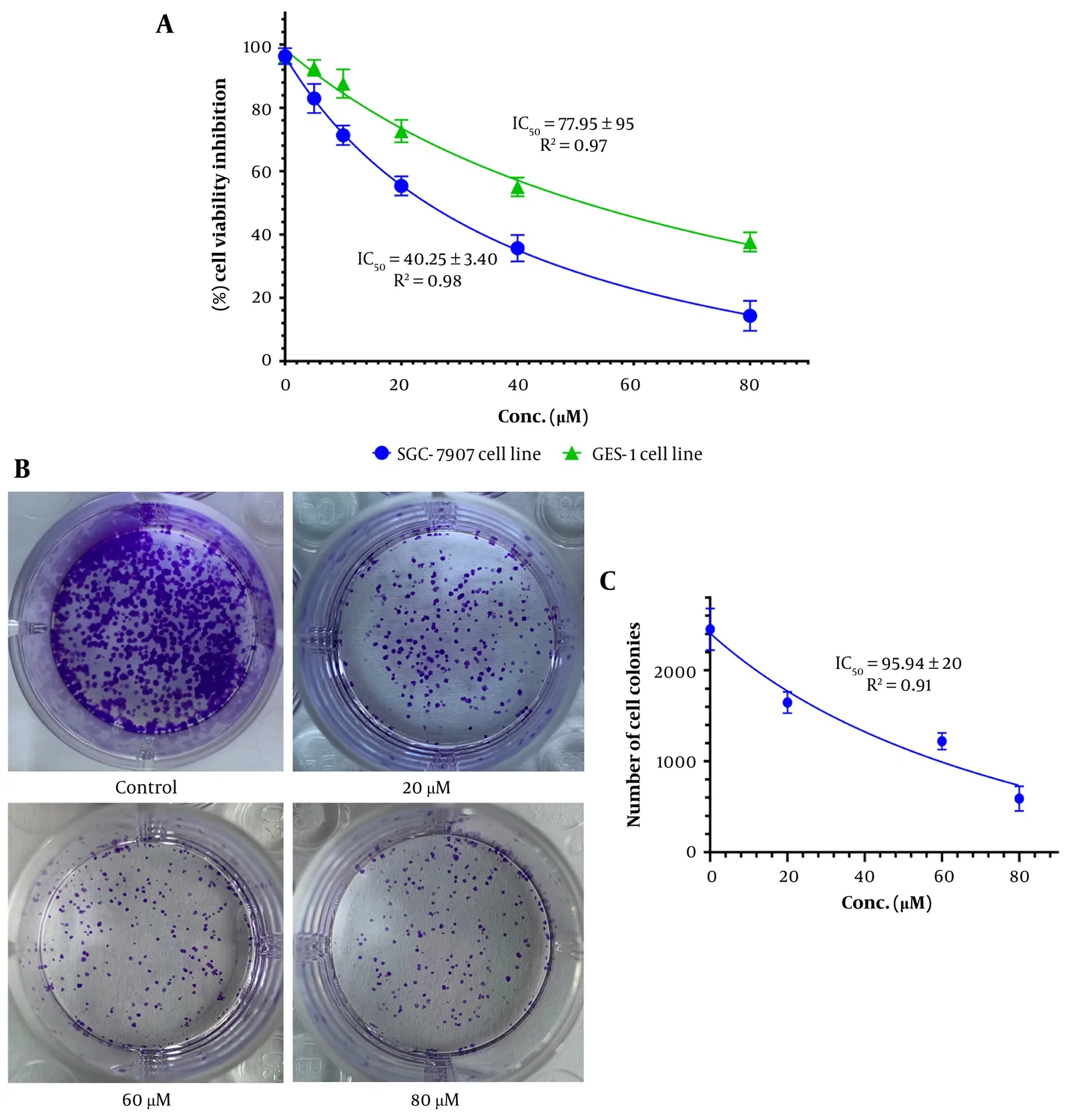
A, MTT assay demonstrating dose-dependent cytotoxicity of baicalein in SGC7901 cells with higher IC_50_ in normal GES-1 cells, indicating selective anticancer activity. B, Representative colony formation images showing progressive suppression of clonogenic potential. C, Quantitative analysis confirming dose-dependent inhibition of colony formation.

### 4.13. Morphological Changes in SGC-7901 Cells After Baicalein Treatment

Morphological assessments showed apoptotic morphology in SGC-7901 cells (Figure 13A). Control cells appeared healthy and adherent; 20 μM baicalein caused cell rounding and partial detachment; and 60 to 80 μM baicalein induced shrinkage, membrane blebbing, vacuolization, and loss of adherence, consistent with dose-dependent apoptosis.

**Figure 13.**
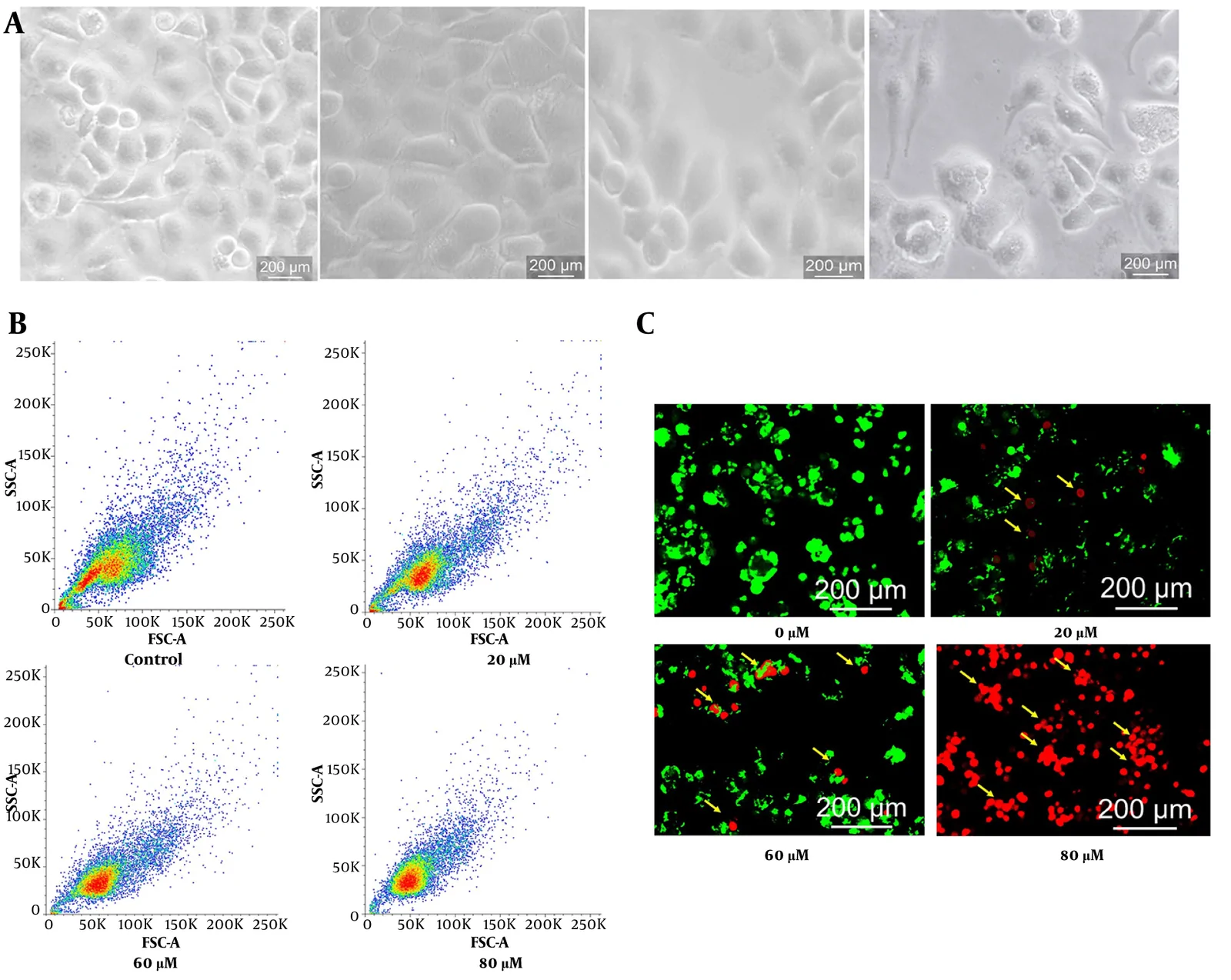
A, Phase-contrast microscopy revealing dose-dependent apoptotic morphological changes, including cell shrinkage, detachment, and membrane blebbing. B, Flow cytometric FSC/SSC analysis indicating progressive reduction in cell size and complexity consistent with apoptosis. C, AO/EB staining confirming increased apoptotic populations with chromatin condensation, fragmentation, and late-stage apoptotic features.

### 4.14. Baicalein-Induced Apoptosis in SGC-7901 Cells

Apoptosis analysis using Annexin V-FITC/PI showed dose-dependent FSC-A/SSC-A changes (Figure 13B). Control cells showed dense viable populations, whereas baicalein caused reduced FSC-A, indicating shrinkage, tighter clusters, and increased debris. At 80 μM, a compact low-FSC population with low viability appeared. Acridine orange/ethidium bromide staining (Figure 13C) confirmed apoptosis. Control cells were green and viable; 20 μM baicalein showed chromatin fragmentation; and 60 to 80 μM baicalein showed orange/red cells with condensation and membrane blebbing, consistent with late apoptosis or necrosis.

### 4.15. Baicalein-Induced G2/M Phase Arrest

Cell cycle analysis showed baicalein-induced effects on cell-cycle distribution. DNA histograms retained a G1 peak with minimal change at low doses (Figure 14A), but quantification (Figure 14B) revealed a stable G0/G1 phase distribution of 50% to 55%, decreased S phase from 42% to 30%, and increased G2/M phase from approximately 5% to 22% at 80 μM. These findings indicate G2/M arrest and reduced GC-cell proliferation.

**Figure 14.**
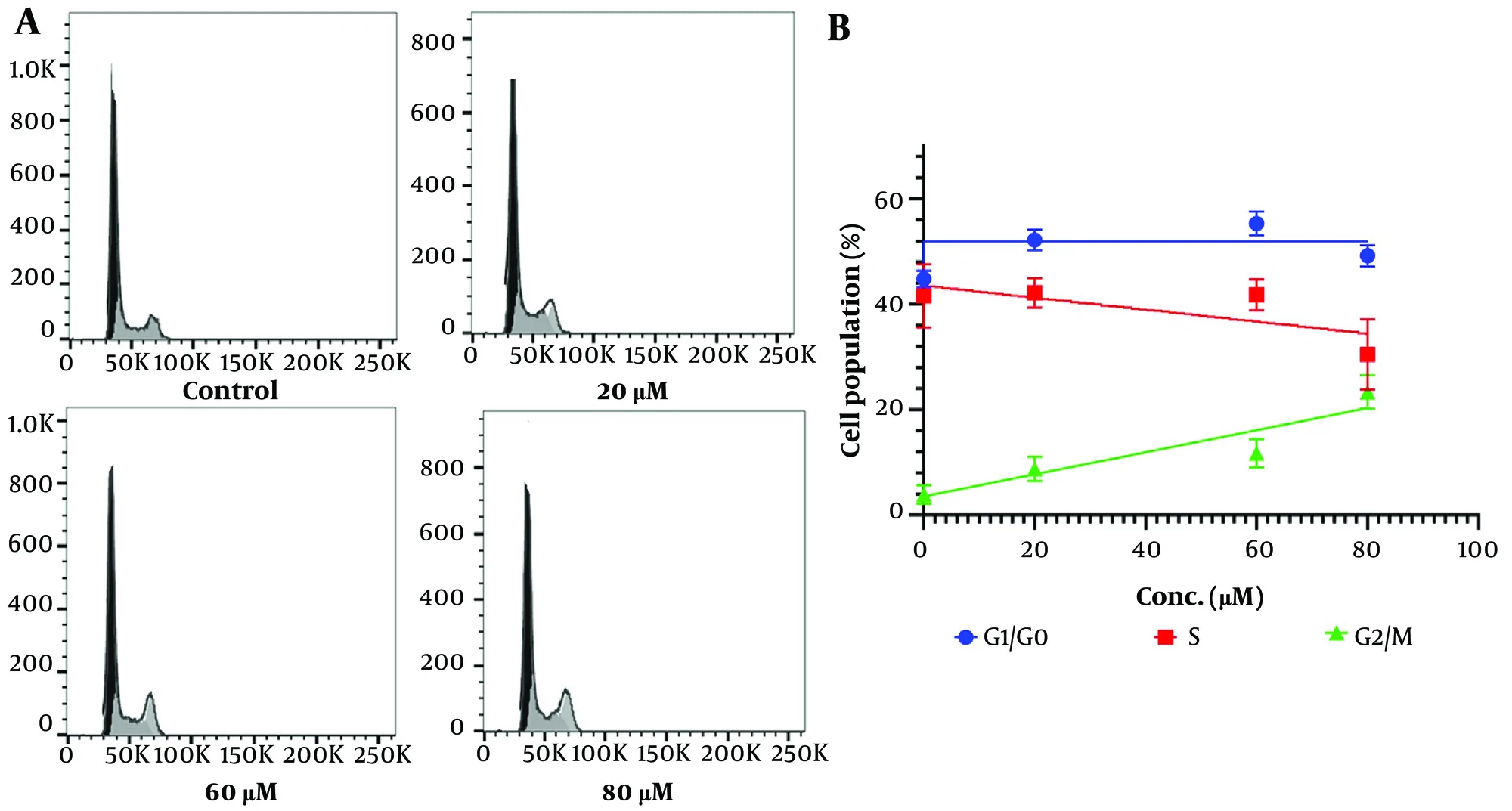
A, Representative DNA-content histograms illustrating cell cycle distribution following baicalein treatment. B, Quantitative analysis demonstrating dose-dependent accumulation of cells in the G2/M phase with reduction in S-phase population, indicating cell cycle arrest and inhibition of proliferation.

### 4.16. Expression of Hub Genes in SGC-7901 Cells After Baicalein Treatment

Western blot analysis showed that baicalein treatment for 24 hours induced dose-dependent downregulation of survival proteins in SGC-7901 cells (Figure 15A). Phosphorylated and total AKT1 and STAT3 levels decreased markedly with increasing concentrations (Figure 15B), along with a progressive reduction in TP53. β-Actin remained unchanged, confirming equal loading. These results suggest that baicalein may inhibit gastric cancer cell survival by suppressing AKT1/STAT3 signaling and modulating TP53.

**Figure 15.**
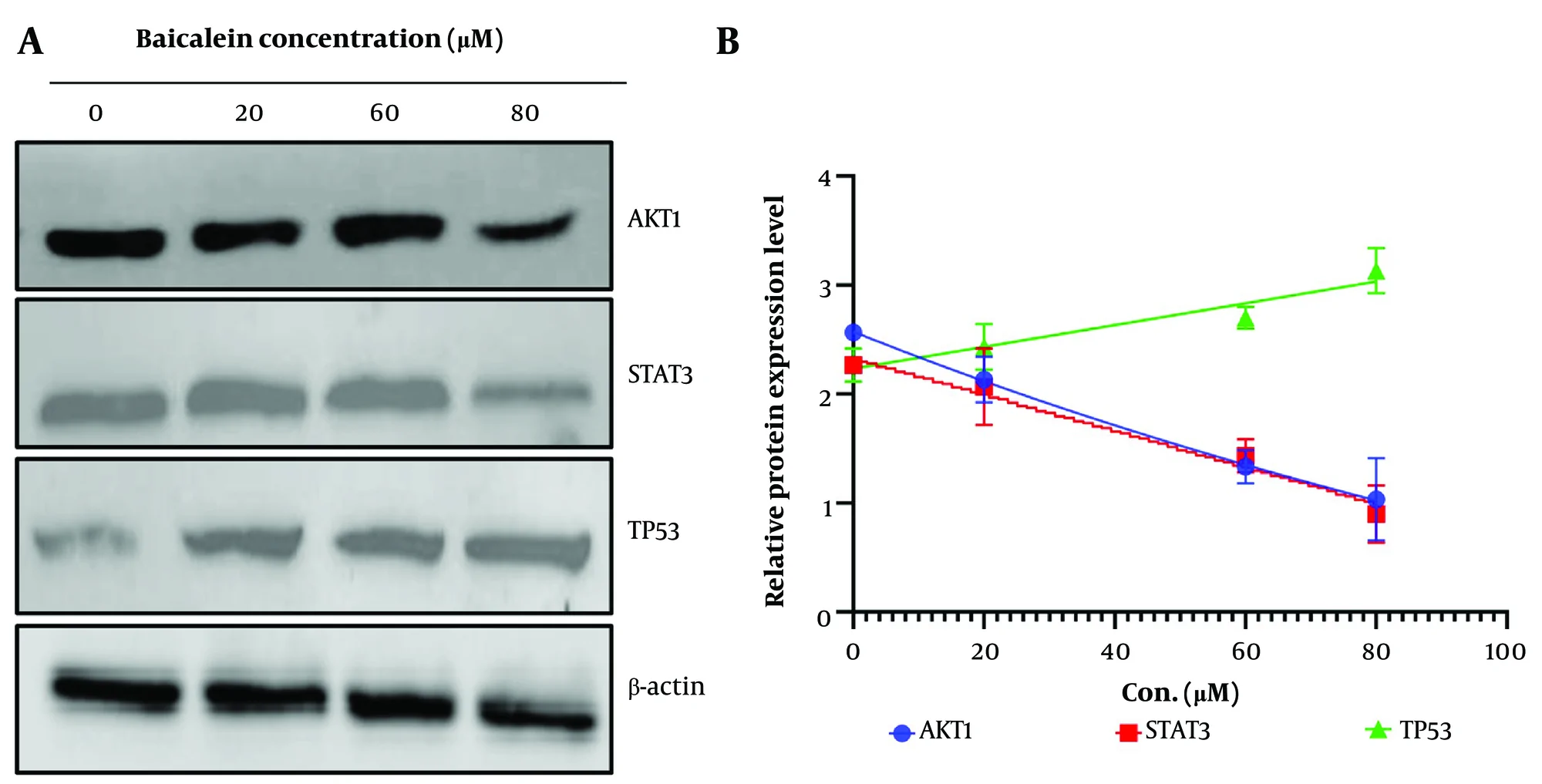
A, Western blot analysis showing dose-dependent downregulation of mutantTP53, AKT1, and STAT3 protein expression in SGC-7901 cells following baicalein treatment. B, Densitometric quantification normalized to β-actin confirming suppression of key oncogenic signaling pathways, supporting the proposed molecular mechanism of anticancer activity.

## 5. Discussion

Taken together, these findings suggest that baicalein may be structurally suited to interact with key oncogenic proteins and may influence cancer cell survival through multiple pathways. Density functional theory analysis suggested that baicalein may interact effectively with biological targets; its moderate HOMO-LUMO gap and electrophilicity indicate balanced stability and reactivity. Oxygen-localized electron density predicted hydrogen bonding, consistent with docking results showing stable interactions with TP53, AKT1, and STAT3. These findings support the predicted binding behavior and align with reports that oxygen-rich flavonoids preferentially bind kinase domains and transcription factors through hydrogen bonding and π-interactions in ATP-binding pockets and SH2 domains (27). Pharmacokinetic and toxicity predictions indicated a favorable drug-like profile, including compliance with all drug-likeness rules, high gastrointestinal absorption, no predicted systemic toxicity, and suitable lipophilicity and TPSA for membrane permeability and solubility. These features align with evidence that such flavonoids show improved uptake and efficacy (27), are consistent with the experimental observation of selective GC-cell inhibition, and suggest a therapeutic window.

The network pharmacology results suggested potential system-level sites of action. Among the predicted targets, 181 overlapped with gastric cancer, indicating a multitarget profile. Protein-protein interaction analysis identified TP53, AKT1, and STAT3 as key hubs regulating tumor growth. AKT1 is associated with survival signaling, STAT3 with proliferation and immune evasion, and TP53 with cell-cycle regulation and apoptosis. Their central roles, particularly sustained AKT and STAT3 signaling in GC, are well supported (28). Enrichment analysis indicated significant associations with the PI3K-Akt, MAPK, and apoptosis pathways, suggesting that baicalein may influence key signaling cascades involved in cancer cell survival. These pathways are linked to proliferation, oxidative stress, and apoptosis. This prediction aligns with evidence that baicalein induces GC-cell death by suppressing the PI3K/AKT pathway and triggering endoplasmic reticulum stress, thereby reducing proliferation and increasing apoptosis (29), supporting PI3K-Akt as a key target pathway.

Gene expression and survival analyses suggested that TP53, AKT1, and STAT3 are upregulated in GC and may be associated with poorer survival outcomes. These findings indicate that the selected targets are potentially relevant to disease progression. TIMER analysis suggested an association between STAT3 and immune cell infiltration, consistent with its reported role in tumor-immune interactions (30).

Docking results suggested favorable binding interactions, with the highest affinity observed for AKT1, followed by STAT3 and TP53. Interaction patterns, including hydrogen bonds, π-interactions, and van der Waals contacts, matched the DFT-predicted reactive sites. Strong AKT1 binding is notable given its central role in cancer survival and is consistent with reports that baicalein inhibits kinase signaling, including JAK2/STAT3 suppression and reduced downstream activity (31), supporting its role as a direct inhibitor of oncogenic proteins.

These predictions align with the in vitro results. Baicalein reduced SGC-7901 viability with lower toxicity in GES-1 cells, indicating selectivity. Colony assays confirmed long-term antiproliferative effects, consistent with prior GC studies (32). Apoptosis and morphological changes, including cell shrinkage, chromatin condensation, and membrane blebbing, indicated cell death and were confirmed by flow cytometry and acridine orange/ethidium bromide staining. This matches pathway predictions, as AKT/STAT3 inhibition promotes apoptosis. Baicalein has also been reported to induce ferroptosis and apoptosis through STAT3 inhibition. Cell-cycle analysis showed G2/M arrest, consistent with PI3K-Akt/MAPK disruption (33) and similar reports (34). Western blotting confirmed dose-dependent downregulation of AKT1 and STAT3 and reduced mutant TP53, supporting pathway inhibition. These findings align with evidence that dual AKT/STAT3 targeting enhances anticancer effects (35). The present findings are also consistent with previous reports on plant-derived compounds, such as *Brassica oleracea*, resveratrol, and *Allium colchicifolium* flavonoids, which demonstrate anticancer effects in gastric cancer models. Similar to these studies, baicalein inhibited proliferation, induced apoptosis, and modulated key signaling pathways, supporting a multitarget mechanism of action (36-38).

AKT1, STAT3, and TP53 are key regulators of GC progression, metastasis, immune evasion, and therapy resistance, making them major targets for precision therapy (39). AKT1, a core kinase of the PI3K/AKT/mTOR pathway, is activated by PI3K amplification, PTEN loss, HER2/EGFR signaling, cytokines, and *Helicobacter pylori* infection. It promotes proliferation, mTOR-mediated protein synthesis, epithelial-mesenchymal transition, angiogenesis, metastasis, antiapoptosis, and resistance to cisplatin, oxaliplatin, trastuzumab, and fluoropyrimidines (39). Therefore, inhibitors including capivasertib, ipatasertib, MK-2206, buparlisib, and everolimus are under investigation. STAT3, activated through IL-6/JAK, EGFR, SRC kinases, inflammatory cytokines, and chronic *H. pylori* infection, induces Cyclin D1, c-Myc, BCL-XL, Survivin, and MCL1, thereby promoting proliferation, survival, epithelial-mesenchymal transition, stemness, angiogenesis, metastasis, immune suppression, and resistance to chemotherapy, targeted therapy, and immune checkpoint blockade (40). STAT3 also enhances PD-L1 expression, inhibits dendritic cells, and recruits regulatory T cells, linking inflammation with immune evasion (40, 41). Consequently, STAT3 inhibitors such as Stattic, napabucasin, OPB-31121, and OPB-51602, JAK inhibitors such as ruxolitinib and tofacitinib, and phytochemicals such as curcumin, quercetin, cucurbitacin B, resveratrol, and EGCG are being explored. TP53, which encodes the tumor suppressor p53, regulates DNA repair, apoptosis, senescence, and cell-cycle arrest, but is mutated in 40% to 60% of GC cases (37, 42). TP53 mutations and loss of heterozygosity cause genomic instability, defective apoptosis, epithelial-mesenchymal transition, metastasis, stemness, immune evasion, and poor response to chemotherapy, pembrolizumab, and adjuvant therapy through loss of p21, BAX, PUMA, and caspase activation (37, 43). Therapeutic strategies include APR-246, COTI-2, WEE1/ATR/CHK1-targeted synthetic lethality, gene restoration, and biomarker-guided immunotherapy (43). Importantly, AKT1, STAT3, and TP53 form an interconnected oncogenic network in which AKT activates STAT3, STAT3 suppresses p53-mediated apoptosis, and TP53 loss enhances PI3K/AKT signaling through PTEN dysregulation. Together, these processes drive proliferation, angiogenesis, epithelial-mesenchymal transition, metastasis, stemness, chemoresistance, and immune escape (37, 39-43). Therefore, combined targeting of AKT1 and STAT3, together with restoration of TP53 function or immune checkpoint blockade, represents a promising strategy for personalized GC therapy.

### 5.1. Limitations

This study has several limitations. The findings are based on in vitro cell line models without in vivo validation, which may limit physiological relevance. Target identification relied on database-driven predictions and was not confirmed by direct biochemical target-engagement assays. In addition, pathway involvement was inferred from expression and association analyses without functional validation using knockdown or rescue studies. Therefore, the translational applicability of these findings remains limited and requires further in vivo and mechanistic investigation.

### 5.2. Conclusions

This study suggests a link between the molecular properties of baicalein and its potential anticancer activity in gastric cancer. Density functional theory and electronic structure analyses showed that baicalein has favorable stability, reactivity, electron delocalization, and charge distribution comparable to established anticancer reference compounds, supporting its strong multitarget interaction potential against AKT1, STAT3, and mutant TP53. Network pharmacology and enrichment analyses suggested modulation of cancer pathways, especially PI3K-Akt and MAPK, indicating a multitarget mode of action. In vitro, baicalein reduced proliferation, increased apoptosis, induced G2/M arrest, and modulated mutant TP53, AKT1, and STAT3 expression. Collectively, these findings suggest potential anticancer effects through multiple survival and apoptotic pathways, although causality remains unconfirmed and requires further validation. Baicalein therefore emerges as a promising candidate for further preclinical and clinical study.

ijpr-25-1-171172-s001.pdf

## Data Availability

The dataset presented in the study is available on request from the corresponding author during submission or after publication.

## References

[1] Sekiguchi M, Oda I, Matsuda T, Saito Y (2022). Epidemiological trends and future perspectives of gastric cancer in Eastern Asia. Digestion.

[2] Sundar R, Nakayama I, Markar SR, Shitara K, van Laarhoven HWM, Janjigian YY (2025). Gastric cancer. Lancet.

[3] Kamandi M, Feiz Disfani H (2025). The role of rs10811661 polymorphism in the CDKN2A/B gene: A narrative review of prognostic biomarker for colon and gastric cancers. Zahedan J Res Med Sci.

[4] Farhan M, Rizvi A, Aatif M, Ahmad A (2023). Current understanding of flavonoids in cancer therapy and prevention. Metabolites.

[5] Wu JY, Tsai KW, Li YZ, Chang YS, Lai YC, Laio YH (2013). Anti-bladder-tumor effect of baicalein from Scutellaria baicalensis Georgi and its application in vivo. Evid Based Complement Altern Med.

[6] Liu H, Dong Y, Gao Y, Du Z, Wang Y, Cheng P (2016). The fascinating effects of baicalein on cancer: A review. Int J Mol Sci.

[7] Yang Y, Hu Y, Gu HY, Lu N, Liu W, Qi Q (2008). Oroxylin A induces G2/M phase cell-cycle arrest via inhibiting Cdk7-mediated expression of Cdc2/p34 in human gastric carcinoma BGC-823 cells. J Pharm Pharmacol.

[8] Kang SY, Hwang D, Shin S, Park J, Kim M, Rahman MH (2021). Potential of bioactive food components against gastric cancer: Insights into molecular mechanism and therapeutic targets. Cancers (Basel).

[9] Adelusi TI, Oyedele AQK, Boyenle ID, Ogunlana AT, Adeyemi RO, Ukachi CD (2022). Molecular modeling in drug discovery. Inform Med Unlocked.

[10] Rosati D, Palmieri M, Brunelli G, Morrione A, Iannelli F, Frullanti E (2024). Differential gene expression analysis pipelines and bioinformatic tools for the identification of specific biomarkers: A review. Comput Struct Biotechnol J.

[11] Varvia P, Räty J, Packalen P (2023). mgpr: An R package for multivariate Gaussian process regression. SoftwareX.

[12] Daina A, Michielin O, Zoete V (2017). SwissADME: A free web tool to evaluate pharmacokinetics, drug-likeness and medicinal chemistry friendliness of small molecules. Sci Rep.

[13] Banerjee P, Kemmler E, Dunkel M, Preissner R (2024). ProTox 3.0: A webserver for the prediction of toxicity of chemicals. Nucleic Acids Res.

[14] Davis AP, Grondin CJ, Johnson RJ, Sciaky D, Wiegers J, Wiegers TC (2021). Comparative toxicogenomics database (CTD): Update 2021. Nucleic Acids Res.

[15] Wei Y, Zhang S, Shang S, Zhang B, Li S, Wang X (2016). SEA: A super-enhancer archive. Nucleic Acids Res.

[16] Daina A, Michielin O, Zoete V (2019). SwissTargetPrediction: Updated data and new features for efficient prediction of protein targets of small molecules. Nucleic Acids Res.

[17] Gallo K, Goede A, Preissner R, Gohlke BO (2022). SuperPred 3.0: Drug classification and target prediction-a machine learning approach. Nucleic Acids Res.

[18] Safran M, Rosen N, Twik M, BarShir R, Stein TI, Dahary D (2022). The GeneCards suite.

[19] Bardou P, Mariette J, Escudié F, Djemiel C, Klopp C (2014). jvenn: An interactive Venn diagram viewer. BMC Bioinformatics.

[20] Syed Zameer Ahmed S, Vetrivel M, Khader SZA, Ragunathan YT, Kumar SK, Prabhu P (2024). Exploring gene network and protein interaction analysis of neurotrophin signaling pathway in ameloblastoma. In Silico Pharmacol.

[21] Tang Z, Kang B, Li C, Chen T, Zhang Z (2019). GEPIA2: An enhanced web server for large-scale expression profiling and interactive analysis. Nucleic Acids Res.

[22] Li T, Fan J, Wang B, Traugh N, Chen Q, Liu JS (2017). TIMER: A web server for comprehensive analysis of tumor-infiltrating immune cells. Cancer Res.

[23] Uhlenbrock N, Smith S, Weisner J, Landel I, Lindemann M, Le TA (2019). Structural and chemical insights into the covalent-allosteric inhibition of the protein kinase Akt. Chem Sci.

[24] Emamzadah S, Tropia L, Vincenti I, Falquet B, Halazonetis TD (2014). Reversal of the DNA-binding-induced loop L1 conformational switch in an engineered human p53 protein. J Mol Biol.

[25] Bai L, Zhou H, Xu R, Zhao Y, Chinnaswamy K, McEachern D (2019). A potent and selective small-molecule degrader of STAT3 achieves complete tumor regression in vivo. Cancer Cell.

[26] Liu Y, Yang X, Gan J, Chen S, Xiao ZX, Cao Y (2022). CB-Dock2: Improved protein-ligand blind docking by integrating cavity detection, docking and homologous template fitting. Nucleic Acids Res.

[27] Badhan RK (2006). Modelling the interactions of flavonoids with ATP-binding cassette transporter nucleotide-binding domains [dissertation].

[28] El-Tanani M, Al Khatib AO, Aladwan SM, Abuelhana A, McCarron PA, Tambuwala MM (2022). Importance of STAT3 signalling in cancer, metastasis and therapeutic interventions. Cell Signal.

[29] Cao M, Tang Y, Abedi-Firouzjah R, Chen J (2025). Therapeutic effects and mechanisms of action of baicalein on stomach cancer: A comprehensive systematic literature review. Discov Oncol.

[30] Ploeger C, Schreck J, Huth T, Fraas A, Albrecht T, Charbel A (2022). STAT1 and STAT3 exhibit a crosstalk and are associated with increased inflammation in hepatocellular carcinoma. Cancers (Basel).

[31] Morshed AKMH, Paul S, Hossain A, Basak T, Hossain MS, Hasan MM (2023). Baicalein as promising anticancer agent: A comprehensive analysis on molecular mechanisms and therapeutic perspectives. Cancers (Basel).

[32] Tian J, Liu C, Li B, Hu N, Gu X, Li D (2025). PAR1 inhibition sensitizes HPV-negative HNSCC cells to ferroptosis through inhibition of the STAT3-mediated regulation of iron and lipid metabolic pathways. Oncogene.

[33] Matthews HK, Bertoli C, de Bruin RAM (2022). Cell cycle control in cancer. Nat Rev Mol Cell Biol.

[34] Bharadwaj KK, Rabha B, Ahmad I, Mathew SP, Bhattacharjee CK, Jaganathan BG (2024). Rhamnetin, a nutraceutical flavonoid arrests cell cycle progression of human ovarian cancer (SKOV3) cells by inhibiting histone deacetylase 2 protein. J Biomol Struct Dyn.

[35] Wang H, Man Q, Huo F, Gao X, Lin H, Li S (2022). STAT3 pathway in cancers: Past, present, and future. MedComm.

[36] Ngo DH, Nguyen HNM, Nguyen TNH, Nguyen TLT, Ngo DN, Vo TS (2022). Growth inhibitory activity of Brassica oleracea var. alboglabra on human gastric cancer cells. J Rep Pharm Sci.

[37] Ghorbani H, Amini K, Jamali B, Tarighi S, Nabiee M, Esfandi A (2025). Resveratrol enhances chemosensitivity of AGS gastric cancer cells to doxorubicin: Focus on drug resistance mechanisms. Compr Health Biomed Stud.

[38] Majnooni MB, Ghanadian M, Mansouri K, Bahrami GR, Mojarrab M (2025). Unveiling the bioactive potential of Allium colchicifolium Boiss bulb flavonoids: Anti-cancer activities, and computational exploration of anti-angiogenic mechanisms. Iran J Pharm Res.

[39] Morgos DT, Stefani C, Miricescu D, Greabu M, Stanciu S, Nica S (2024). Targeting PI3K/AKT/mTOR and MAPK signaling pathways in gastric cancer. Int J Mol Sci.

[40] Zhao C, Li H, Lin HJ, Yang S, Lin J, Liang G (2016). Feedback activation of STAT3 as a cancer drug-resistance mechanism. Trends Pharmacol Sci.

[41] Yu H, Lee H, Herrmann A, Buettner R, Jove R (2014). Revisiting STAT3 signalling in cancer: New and unexpected biological functions. Nat Rev Cancer.

[42] Kim M, Seo AN (2022). Molecular pathology of gastric cancer. J Gastric Cancer.

[43] Tornesello M (2024). TP53 mutations in cancer: Molecular features and therapeutic opportunities. Int J Mol Med.

