# Supplementary material for: Deciphering the Anticancer Effects of Baicalein in Human Gastric Cancer Cells: Computational Chemistry, Bioinformatics, Network Pharmacology Insights, and in vitro Experimental Validation
Source: Iran J Pharm Res. 2026 Jun 16;25(1):e171172. doi: 10.5812/ijpr-171172 (PMC13769050; doi:10.5812/ijpr-171172)
Supplement: Supplementary file 1 [file ijpr-25-1-171172-s001.pdf]

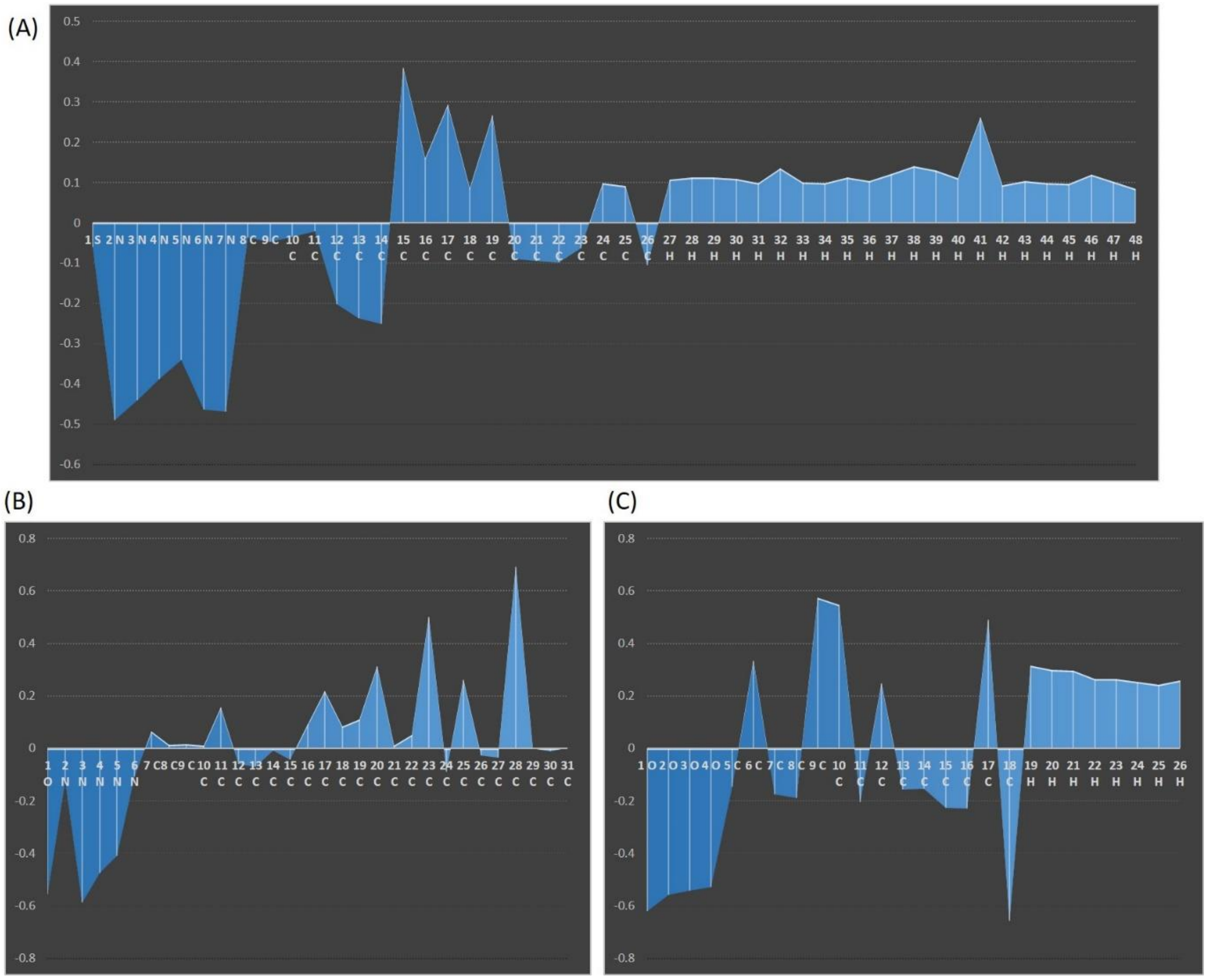

(A)
0.5
0.4
0.3
0.2
0.1
0
-0.1
-0.2
-0.3
-0.4
-0.5
-0.6
1 S 2 N 3 N 4 N 5 N 6 N 7 N 8 C 9 C 10 C 11 C 12 C 13 C 14 C 15 C 16 C 17 C 18 C 19 C 20 C 21 C 22 C 23 C 24 C 25 C 26 C 27 H 28 H 29 H 30 H 31 H 32 H 33 H 34 H 35 H 36 H 37 H 38 H 39 H 40 H 41 H 42 H 43 H 44 H 45 H 46 H 47 H 48 H
(B)
0.8
0.6
0.4
0.2
0
-0.2
-0.4
-0.6
-0.8
1 O 2 N 3 N 4 N 5 N 6 N 7 C 8 C 9 C 10 C 11 C 12 C 13 C 14 C 15 C 16 C 17 C 18 C 19 C 20 C 21 C 22 C 23 C 24 C 25 C 26 C 27 C 28 C 29 C 30 C 31 C
(C)
0.8
0.6
0.4
0.2
0
-0.2
-0.4
-0.6
-0.8
1 O 2 O 3 O 4 O 5 C 6 C 7 C 8 C 9 C 10 C 11 C 12 C 13 C 14 C 15 C 16 C 17 C 18 C 19 H 20 H 21 H 22 H 23 H 24 H 25 H 26 H
